# A Systematic Review and Meta-Analysis of Within-Person Changes in Cardiac Vagal Activity across the Menstrual Cycle: Implications for Female Health and Future Studies

**DOI:** 10.3390/jcm8111946

**Published:** 2019-11-12

**Authors:** Katja M. Schmalenberger, Tory A. Eisenlohr-Moul, Lena Würth, Ekaterina Schneider, Julian F. Thayer, Beate Ditzen, Marc N. Jarczok

**Affiliations:** 1 Institute of Medical Psychology, Center for Psychosocial Medicine, University Hospital Heidelberg, 69115 Heidelberg, Germany; lena.wuerth@posteo.de (L.W.); ekaterina.schneider@med.uni-heidelberg.de (E.S.); beate.ditzen@med.uni-heidelberg.de (B.D.); 2 Women’s Mental Health Research Program, Department of Psychiatry, University of Illinois at Chicago, Chicago, IL 60612, USA; t.eisenlohr.moul@gmail.com; 3 Department of Psychological Science, School of Social Ecology, University of California Irvine, Irvine, CA 92697-7085, USA; 4 Clinic for Psychosomatic Medicine and Psychotherapy, Ulm University Medical Center, 89081 Ulm, Germany

**Keywords:** female health, menstrual cycle, ovarian hormones, cardiac vagal activity, heart rate variability, cardiac vagal tone

## Abstract

Interest in cardiac vagal activity (CVA; e.g., parasympathetically-mediated heart rate variability) as a biomarker of physical and mental health has increased exponentially in recent years. However, the understanding of sources of within-person change (i.e., intra-individual variance) in CVA is lagging behind. This systematic review and meta-analysis summarizes and quantifies current empirical evidence of within-person changes in measures of CVA across the menstrual cycle in naturally-cycling premenopausal females. We conducted an extensive literature search following the Preferred Reporting Items for Systematic Reviews and Meta-Analyses (PRISMA) statement in five databases to identify observational studies with repeated measures of CVA in at least two menstrual cycle phases. A broad meta-analysis (*n*_studies_ = 37; *n*_individuals_ = 1,004) revealed a significant CVA decrease from the follicular to luteal phase (*d* = −0.39, 95% CI (−0.67, −0.11)). Furthermore, 21 studies allowed for finer-grained comparisons between each of two cycle phases (menstrual, mid-to-late follicular, ovulatory, early-to-mid luteal, and premenstrual). Significant decreases in CVA were observed from the menstrual to premenstrual (*n*_studies_ = 5; *n*_individuals_ = 200; *d* = −1.17, 95% CI (−2.18, −0.17)) and from the mid-to-late follicular to premenstrual phases (*n*_studies_ = 8; *n*_individuals_ = 280; *d* = −1.32, 95% CI (−2.35, −0.29)). In conclusion, meta-analyses indicate the presence of CVA fluctuations across the menstrual cycle. Future studies involving CVA should control for cycle phase. Recommendations for covarying or selecting cycle phase are provided.

## 1. Introduction

Cardiac vagal activity (CVA) describes the innervation of the heart mediated by the restorative parasympathetic branch of the autonomic nervous system (i.e., the vagus nerve). In recent years, a large number of studies have investigated the association between CVA and mental health, identifying CVA as a transdiagnostic biomarker for psychological and psychiatric functioning [1]. Since decreased levels of CVA are associated with poorer emotional regulation [2,3], cognitive control, resilience, adaptability [4], and social engagement [5], CVA reductions have been connected to depression and anxiety [6,7,8,9], even in healthy populations [10]. The neurovisceral integration model [11] suggests that this goes back to CVA being mainly generated by interdependent regulatory systems of the central-autonomic network (CAN), including cortical components (e.g., the medial prefrontal, anterior cingulate, and insular cortex) and several nuclei (e.g., the paraventricular nucleus and amygdala), which in turn, also support emotional and cognitive self-regulation. Therefore, according to the neurovisceral integration model, a high connectivity and functional capacity of these CAN areas is reflected in both higher levels of CVA and better psychological and psychiatric functioning, explaining CVA’s role as a biomarker for psychopathology.

Next to this association between CVA and mental health, decades of research have also identified the high relevance of CVA to physical health. Due to its association with peripheral immune dysregulation and inflammation [12], as well as glucose dysregulation [13], decreased levels of CVA have been connected to a higher risk of cardiovascular disease [14,15], obesity, metabolic syndrome and type 2 diabetes mellitus [16], rheumatoid arthritis [17,18], and certain types of cancers [19].

A broad range of studies have identified a multitude of factors contributing to interindividual (i.e., between-person) variability in CVA; for example, biological sex [20]; genetic factors, like the COMT Val158Met genotype [21]; medical factors, like obesity [22] and medication use (e.g., analgesic intake) [23]; life style factors, such as alcohol intake [24], drug use [25,26], and physical activity [27]; and environmental factors such as stress at work [28]. In contrast, research on intraindividual (i.e., within-person) variability in CVA is less extensive. For example, CVA levels fluctuate intraindividually in a circadian manner, with CVA levels peaking during the night [29]; in a physical-exercise-dependent manner, with CVA levels decreasing during exercise and gradually increasing within the first minutes of recovery [30,31]; and in a mental stress-dependent manner, with CVA levels being lower during acute mental stress sessions than at rest [32].

One gender-specific factor investigated for its association with intraindividual variations in CVA is the menstrual cycle. Menstrual cycle-related varying CVA levels might be associated with fluctuations of emotional and cognitive self-regulatory capacity, and consequently, have implications for females’ daily functioning and well-being. Throughout the female reproductive lifespan, which lasts from menarche to menopause for an average of 36 years [33], the menstrual cycle is a constantly repeated natural process of the reproductive system. Typical menstrual cycles in healthy females last an average of 28 days (with 21 to 35 days considered normal) and are characterized by predictable fluctuations of the ovarian hormones estradiol (E2) and progesterone (P4). Based on menstrual onset and ovulation, the menstrual cycle can broadly be divided into two phases: the follicular phase, starting with the onset of menses and lasting until the end of ovulation (of variable length between and within individuals, usually lasting anywhere from 7 to 21 days), and the luteal phase, beginning right after ovulation and ending with the day before the subsequent onset of menses (fixed at around 14 days due to the predictable lifespan of the corpus luteum [34]). The follicular phase is characterized by consistently low levels of P4 and a gradual rise in E2, punctuated by a prominent pre-ovulatory E2 surge that peaks just prior to ovulation. Right after ovulation, P4 levels start to rise, peaking in the mid-luteal phase about one week after ovulation. E2 levels also show a secondary, smaller mid-luteal peak at that time. Both E2 and P4 levels demonstrate rapid withdrawal in the second half of the premenstrual week, leading to generally low levels around the onset of the subsequent menses, and the cycle begins again [33]. Since the luteal phase has about the same length in most of the naturally-cycling females, variations in cycle length between and within females are caused by variations in the length of the follicular phase [34].

E2 and P4 affect a wide array of brain functions, including emotional processing and cognitive functioning [35], mediated through the high density of E2 and P4 receptors in brain areas, such as the hypothalamus, limbic system and prefrontal cortex [36,37,38]. Since some of these areas also belong to the CAN which regulates CVA [11], the menstrual cycle may impact the functioning of the CAN and induce fluctuations in CVA across the cycle. However, findings on the association between the menstrual cycle and within-person variations of CVA are not only inconsistent but contradictory. With this systematic review and meta-analysis, we aim to summarize and quantify the existing empirical evidence regarding the association of the menstrual cycle with parasympathetic innervation of the heart. In addition, potential reasons for inconsistent findings are systematically evaluated and recommendations for future studies are provided. The results and recommendations would not only be relevant for female physical and mental health research, but also for future studies in which CVA is measured in samples with naturally-cycling females of physically fertile age.

## 2. Methods

### 2.1. Literature Search and Screening Criteria

**Figure 1:**
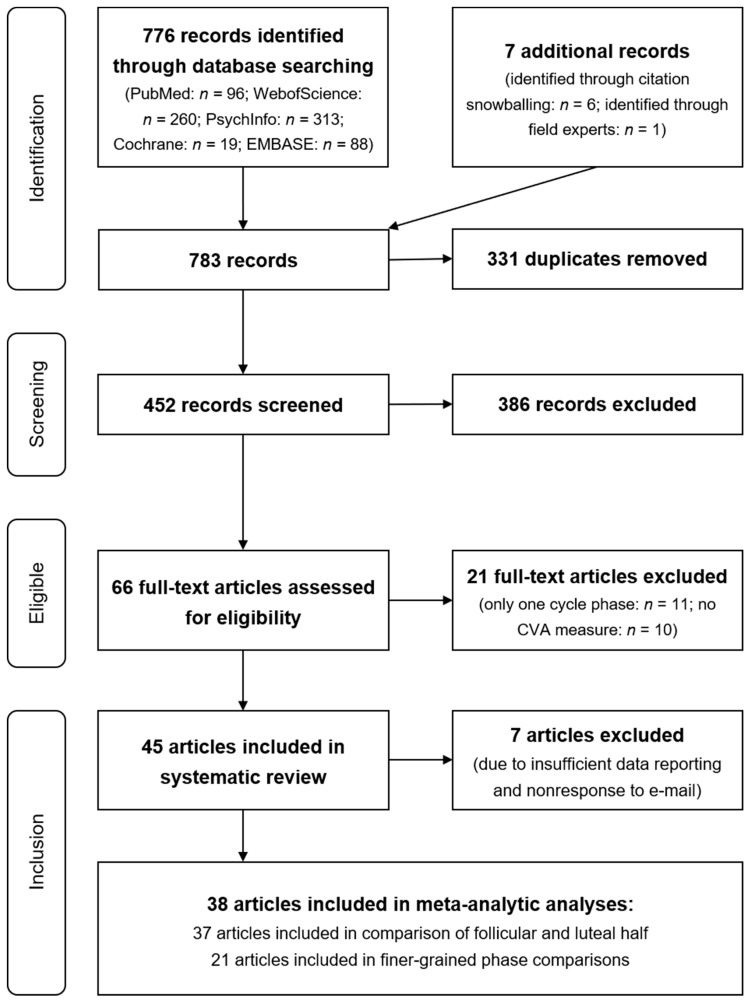
Prisma flow chart depicting the flow of information through the different phases of the systematic review and meta-analysis.

A systematic search of the literature, according to the Preferred Reporting Items for Systematic Reviews and Meta-Analyses (PRISMA) statement [39], was employed and is displayed in Figure 1. A total of five databases (PubMed, WebOfScience, PsychInfo, Cochrane, and Embase) were screened for studies reporting within-person changes in CVA across the menstrual cycle in naturally-cycling adult females. Medical subject heading (MesH) or a comparable method was used to identify search terms (see Appendix A, for detailed search strategy by database).

The initial search was conducted in July 2016 by the authors L.W., M.N.J., and K.M.S. and updated by M.N.J. and K.M.S. in October 2018. All empirical studies published up to that date were considered. Database searches yielded 776 records with an additional 7 records identified through citation snowballing and field experts. After removing 331 duplicates that were found in more than one database, 452 records remained.

### 2.2. Study Selection

Studies were considered for inclusion into the systematic review and meta-analysis if all of the following a priori criteria were met:

The study represents original research and was published in English, German, or Russian as an article or brief report in a journal (i.e., no comments, dissertations, and conference abstracts). At least two repeated measures of CVA within the same female in at least two different menstrual cycle phases (menstrual, mid-to-late follicular, ovulatory, early-to-mid luteal, premenstrual) were assessed in premenopausal, naturally-cycling (i.e., no use of hormonal contraception, and not pregnant or breastfeeding) human, adult (i.e., age 18 or older) females.

Studies were included if at least one distinguishable subsample was reported that met the inclusion criteria. For example, if the full text reported a group of premenopausal females in comparison to a group of menopausal females, only data from the first group were extracted. By applying the inclusion criteria to the information contained in the title and abstract, the pool of records was reduced to 66. After reviewing the full text, a total of 45 studies from 45 publications remained.

Abstract screening and selection were carried out independently by the authors L.W., M.N.J., and K.M.S. in July 2016 and by M.N.J. and K.M.S. in October 2018. Potential conflicts were resolved within the group of three in 2016 and the group of two in 2018 until consensus could be reached. Abstracts written in Russian were screened by E.S. When selecting abstracts in 2016, L.W., M.N.J., and K.M.S. reached an overall inter-rater agreement of 92%, while M.N.J. and K.M.S. came to an agreement in 96% of cases in 2018. In both years, full-text selection showed a 100% agreement.

In case of any missing or inconclusive information in the full text, the corresponding author of the publication in question was searched electronically to retrieve their current e-mail address and was then contacted by L.W., M.N.J., and K.M.S. repeatedly with the friendly request to provide the missing or additional information. In case no current address of the corresponding author could be found, the co-authors were approached. Altogether, 33 authors from 28 full-text publications were contacted, of which 22 authors responded concerning 21 of the publications. At this point, we would like to thank all authors who responded for their cooperation.

### 2.3. Data Extraction

The following data was extracted from all studies we included: (I) name of all authors, (II) year of publication, (III) sample size, (IV) potential between-person subsampling of these individuals (e.g., by personality traits, eating habits, physical activity status, etc.), (V) the experimental condition closest to baseline that was chosen for data extraction, (VI) cycle phases that were compared by the authors and their approach for cycle phase determination, (VII) authors’ potential validation of cycle phase, (VIII) the measure indexing CVA, and (IX) body position during the assessment of CVA.

In case authors reported (V) multiple experimental conditions in which they investigated within-person changes of CVA, baseline results (i.e., no intervention) were extracted by default. In the five cases where no such baseline was reported, the following decisions were made: Balayssac-Siransy et al. [40] asked their participants to perform an exercise test and measured heart rate recovery in absolute value for six minutes. In line with standard procedure (e.g., [41]), we retrieved the data from the third recovery minute for the present meta-analysis. De Zambotti et al. [42] assessed menstrual cycle-related variations in CVA during their participants’ rapid eye movement (REM) sleep and during their non-rapid eye movement (NREM) sleep. For the present meta-analysis, the data of the REM sleep phase were used, since the general physiology during REM sleep mimics the physiology in the waking state more closely than the physiology during NREM sleep. Voronova et al. [43] invited their participants in for repeated cycle-dependent measures of CVA, once in the spring and once in the fall. Since autumn in Russia, where data collection took place, is associated with sinking temperatures and light condition, which in turn can be related to a general negative affect, and since emotional functioning is associated with CVA, we decided to extract the spring data for the meta-analysis. Chung and Yang’s [44] sample consisted of nurses who were asked to measure their CVA with a wrist actigraph for 24 h in three different cycle phases during their day, evening and night shifts. Since most other studies recorded the CVA of their subjects during the day, the data from the day shift were included in the meta-analysis for greater comparability. Ohara et al. [45] examined cycle-dependent CVA fluctuations in their participants once in an eating and once in a fasting trial. In both trials, baseline data were collected before eating and fasting began. The baseline data of the eating-trial were included in the meta-analysis, as they are more similar to everyday life in that no fasting was anticipated.

Regarding (VIII) the measure indexing CVA, several measures of vagal activity exist, mostly based on heart rate variability (HRV; i.e., variability in the timing between successive heart beats in a given time interval) or specific test protocols, such as the deep breathing test or Valsalva maneuver. There were no limitations in the inclusion of various indicators of CVA as long as they quantified the parasympathetic innervation of the heart. Since it is the effect size of the within-person variation of CVA in each study that was included in the meta-analysis, and since effect sizes are independent of the corresponding CVA indexes, data with different indices could be aggregated. A definition and summary of vagal activity measures included in the systematic review and meta-analysis is given in Table 1. In some studies, different measures of vagally-mediated HRV are reported (e.g., high frequency band (HF) and root mean squared difference of successive R–R intervals (RMSSD)). If this was the case, RMSSD was always prioritized over HF in data extraction since RMSSD has been shown to be less susceptible to artifacts [46].

Some authors assessed and reported (IX) CVA indices in study participants during multiple body positions. Since, in the majority of studies participants were lying during CVA assessment, it was that data that was always preferred over that from any other body position.

All statistical analyses were performed with Stata 15.1 (SE, Stata Corp. College Station, TX, USA). We used the “metan” command [47] for all meta-analytic calculations and the “metafunnel” command for creating the funnel plot and performing the corresponding Egger test [48].

### 2.4. Cycle Phase Reclassification

Since the authors used different approaches to dividing the cycle into phases, major cycle phase reclassification was necessary in most of the included studies. The following systematic cycle phase classification was applied based on the typically-assumed variations in ovarian hormones: menstrual phase (low E2; low P4), mid-to-late follicular phase (rising E2; low P4), ovulatory phase (peak E2; low P4), early-to-mid luteal phase (secondary lesser peak of E2; peak P4), and premenstrual phase (falling E2; falling P4). For each of the original papers included in the review and meta-analysis, we used the information on cycle phase description given by the authors to relabel the cycle phase during which the CVA assessment was done.

In general, two types of information (day of menstrual onset and day of ovulation) allowed for two different approaches for cycle phase determination (cycle-day-based and ovulation-based; see Table 2 for an overview). Cycle-day-based approaches were used in the present study when information was limited to the date (or dates) of menstrual onset. With the forward-count approach, onset of menses (cycle day 1) was used to characterize the menstrual cycle that followed. Given that under these circumstances no information is reported about the next onset of menses, individualization of cycle length is not possible with this approach, and an average 28-day cycle is assumed in all individuals. For studies in the present review that used the forward-count approach, five cycle phases were defined as follows: cycle days 1–7 (menstrual), 8–12 (mid-to-late follicular), 13–16 (ovulatory), 17–21 (early-to-mid luteal), and 22–28 (premenstrual). In other studies, more information was available for cycle phase assignment. The backward-count approach, for example, is possible when both onset of the prior menses and onset of the following menses are known. This approach starts by counting backward from the next menses onset (such that the day prior to the next menses onset is labeled −1 and so forth) in order to characterize the previous menstrual cycle. As mentioned before, within and between-person differences in cycle length are assumed to be mainly caused by variations in the follicular phase duration, since the luteal phase (from ovulation till onset of menses) is assumed to relatively robustly last 14 days. Therefore, the backward counting method is particularly well suited for determining an individual’s time of ovulation, her early-to-mid luteal phase, and her premenstrual phase. In line with previous literature [55,56], for studies in the present review that provide onset of next menses and onset of prior menses, we characterized three of the cycle phases more precisely: ovulatory (cycle days −15 to −12), early-to-mid luteal (cycle days −11 to −8), and premenstrual (cycle days −7 to −1).

While the forward and backward-count approaches are merely dependent on the onset of menses, ovulation-based approaches require a biological measure for determination of ovulation. Typically, a urine-based test of the luteinizing hormone (LH) level or the measurement of the basal body temperature (BBT) are employed to confirm the timing of ovulation, as these two methods allow for an at home use by the participants (as opposed to, for example, ultrasonography to determine follicle maturation and disappearance [57]). Cycle phase determination based on the LH-testing approach relies on the fact that the LH hormone has a single distinct peak across the menstrual cycle which occurs at the time of ovulation and can be detected by urine-based tests with a typical sensitivity of 10–70 mIU/mL. Since ovulation lasts 12–36 h, the following ovulation-based definition of cycle phases is generally applied: day of and day +1 following positive LH test (ovulatory phase), days +2 to +7 following positive test result (early-to-mid luteal phase), and days +8 to +14 following positive test result (premenstrual phase). The same applies to the BBT-based approach: It requires females to assess their BBT daily in order to register its biphasic temperature change from lower BBT in the follicular and higher BBT in the luteal phase of the cycle. Since the rise in BBT is caused by ovulation, the nadir the day before the temperature rise as well as the first day of the rise are generally assumed to constitute the ovulatory phase. Days +2 to +7 following the nadir represent the early-to-mid luteal phase, and days +8 to +14 following the nadir the premenstrual phase.

Cycle phase reclassification was carried out jointly by two experts (authors T.A.E.-M. and K.M.S.). All information given by the authors of the original publications was used to determine cycle phase according to the just outlined cycle-day and/or ovulation-based approaches. If the authors of the original publications reported a time span (in cycle days) in which they had assessed CVA that covered two of the time spans described above, the CVA assessment was assigned to the cycle phase that comprised the majority of the cycle days reported by the authors.

### 2.5. Identification of Studies with Precise Cycle Phase Determination

An inexact determination of cycle phase causes noise when investigating true CVA fluctuations across the cycle and makes meta-analytic results less meaningful. For this reason, after each cycle phase had been reclassified as outlined above, a binary decision was made as to whether each cycle phase was precisely determined by the authors of the original publication. The following requirements for precise cycle phase determination served as the basis for these decisions:

(I) The menstrual cycle phase and the mid-to-late follicular phase can be precisely determined by the forward-count method.

(II) In order to accurately delineate the ovulatory, early-to-mid luteal, and premenstrual phases, the backward-count method or any method that involves ovulation testing had to be applied.

## 3. Results

### 3.1. Study Characteristics

A total of 45 studies published between the year 1995 and October 2018 were included in the systematic review [40,42,43,44,45,49,50,51,52,53,54,57,58,59,60,61,62,63,64,65,66,67,68,69,70,71,72,73,74,75,76,77,78,79,80,81,82,83,84,85,86,87,88,89,90]. Only one of the 45 studies was published in Russian [43]; all others were written in English. No German-language publication was found meeting the above-mentioned inclusion criteria. Study characteristics extracted from the original studies are listed in Table 3. All details of the publications that remained unspecified are marked with “n/a” (not available) in Table 3. Sample sizes ranged from 6 to 100 with a median of 18 and a mean of 25. Regarding subsampling of participants as a function of interindividual differences, a total of five studies compared healthy females with those meeting prospective criteria for premenstrual syndrome [61] or premenstrual dysphoric disorder [70] or meeting retrospective criteria for premenstrual syndrome [42,53,73]. One study subsampled their participants according to eating habits [63], one study according to athletic state [76], one study according to average heart rate (HR) [83], and one study according to the personality trait neuroticism [72]. A total of four studies assessed within-person change in CVA across the menstrual cycle in different intraindividually changing experimental conditions: Balayssac-Siransy et al. [40] assessed menstrual cycle-related CVA changes in different stages of recovery after exercise, de Zambotti et al. [42] in different sleep phases (NREM versus REM sleep), Voronova et al. [43] in different seasons (spring versus fall), and Chung and Yang [44] in different work shifts (day versus evening versus night shift).

The results of the reclassification process of cycle phases, as outlined above, are listed in Table 3. In each case, it is also indicated (in inverted commas) how the authors of the original publications had initially labeled the cycle phase. After reclassifying the cycle phases of all 45 studies included in the systematic review, the following patterns emerged: CVA was assessed in the menstrual phase in 31 studies (69%), in the mid-to-late follicular phase in 28 studies (62%), in the ovulatory phase in nine studies (20%), in the mid-to-late follicular phase in 26 studies (58%), and in the premenstrual phase in 23 studies (51%). Less than half of the studies (*n*_studies_ = 19; 42%) reported retrospective cycle phase validation via analyses of at least one ovarian hormone level. In 18 of these studies, hormone levels were analyzed via blood samples; in one study, urine samples were used. The most commonly used indicator for CVA was any measure of heart rate variability (HF or RMSSD; *n*_studiess_ = 37; 82%). Most of the 36 studies with available information on body position during CVA assessment reported CVA measurements in the supine position (*n*_studiess_ = 24; 67%).

### 3.2. CVA Change from the Follicular Phase to the Luteal Phase

The broadest division of the menstrual cycle is in its two halves: the follicular phase (from onset of menses until ovulation) and the luteal phase (from the day after ovulation until the day before the onset of the subsequent menses). Regarding hormone levels, the comparison of these two halves can be roughly translated into a comparison of low (follicular) versus high (luteal) hormone levels, especially with regard to P4 levels. Additionally, this broader phasing of the menstrual cycle in only two parts (compared to the five phases defined above) allows for more inclusive meta-analytic calculations, since almost all studies (*n*_studies_ = 44) report a comparison of a phase of the follicular half (i.e., menstrual, mid-to-late follicular, or ovulatory) with a phase of the luteal half (i.e., early-to-mid luteal or premenstrual).

Overall, 38 of the 45 studies (*n*_individuals_ = 1018) included in the systematic review, report quantitative data suitable for meta-analytic calculations [40,42,43,44,45,50,52,53,54,57,58,59,61,62,63,64,65,66,67,68,70,71,72,73,74,75,76,77,78,80,82,84,85,86,87,88,89,90]. Out of these 38 studies, only one study [57] did not compare a phase of the follicular half with a phase of the luteal half, but assessed the menstrual and the ovulatory phase (two follicular phases). The remaining 37 studies reporting quantitative data could be included in the meta-analytic comparison of the follicular and the luteal phase which is based on 1004 females [40,42,43,44,45,50,52,53,54,58,59,61,62,63,64,65,66,67,68,70,71,72,73,74,75,76,77,78,80,82,84,85,86,87,88,89,90].

**Figure 2:**
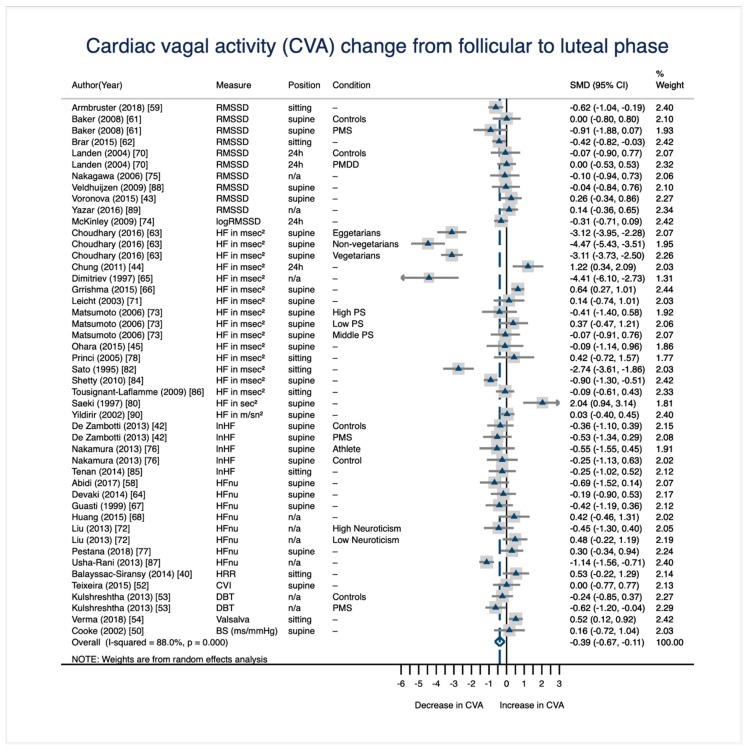
Meta-analytic results on within-person change of CVA from the follicular to the luteal phase of the menstrual cycle (*N*_studies_ = 37; *N*_SMD_ = 47; *N*_individuals_ = 1,004). Note. SMD = standardized mean difference (effect size); RMSSD = root mean square of successive differences between adjacent RR intervals; HF = high frequency component in the power spectrum range; HRR = heart rate reactivity; CVI = cardiac vagal index; DBT = deep breathing test; Valsalva = valsalva ratio; BS = baroreflex slope; n/a = information not available; PMDD = premenstrual dysphoric disorder; PMS = premenstrual syndrome; PS = premenstrual symptomatology.

In case a study had assessed more than one phase of the follicular half, the mid-to-late follicular phase was always preferred over the menstrual phase and the menstrual phase was always preferred over the ovulatory phase when selecting data for the meta-analytic comparison of the follicular and the luteal phase. This is due to the fact that the mid-to-late follicular hormonal profile is most representative for the follicular phase, since the menstrual phase may contain withdrawal of hormones and the ovulatory phase is characterized by a brief, abrupt rise in E2 levels that does not characterize the entire follicular half. If a study had assessed more than one phase of the luteal half, the early-to-mid luteal phase was preferred over the premenstrual phase. This is due to the fact that the early-to-mid luteal testing is more representative of the entire luteal phase in the sense that both hormones E2 and P4 show rising but mostly high levels and no withdrawal (as observable in the premenstrual testing). Across the 37 studies included in the meta-analytic comparison of the follicular and the luteal phase, the follicular phase was represented by the mid-to-late follicular phase in 23 studies and by the menstrual phase in 14 studies. The luteal phase was represented 24 times by the early-to-mid luteal phase and 13 times by the premenstrual phase. The corresponding dataset is provided in the Supplemental Materials. Results of the meta-analysis are displayed in the forest plot in Figure 2. The forest plot was comprised of 37 studies but lists 47 effect sizes (i.e., standardized mean difference; SMD), since nine studies subsampled their participants according to interindividual differences (e.g., personality traits). Each of these conditions (e.g., high versus low neuroticism) is represented by a separate SMD. Overall, there is a significant decrease in CVA from the follicular to the luteal phase of the menstrual cycle that shows a conventionally medium effect size [91] of *d* = −0.39 and a 95% CI of (−0.67, −0.11).

**Figure 3:**
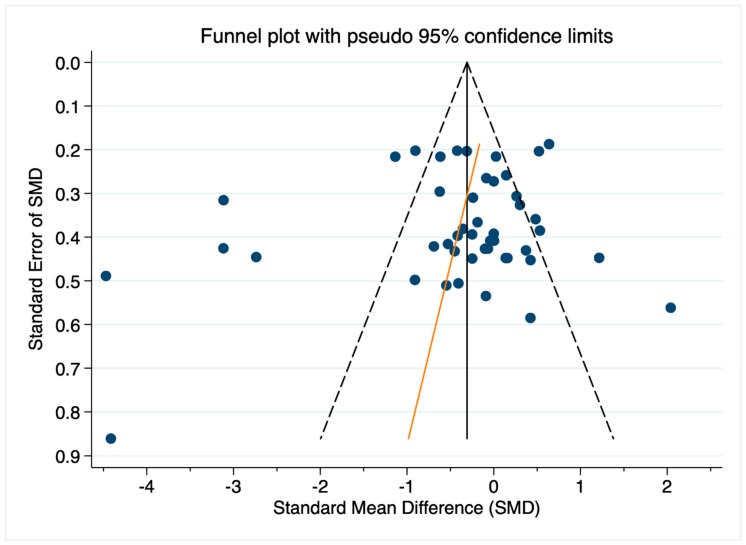
Funnel plot of 47 effect sizes (i.e., standardized mean difference, SMD) from 37 studies on within-person change of CVA from the follicular to the luteal phase. Effect sizes (SMDs) are plotted against the standard error of SMD. The orange line indicates the fitted regression line from the standard regression (Egger) test for small-study effects. Note: SMD < 0 indicates a CVA decrease from the follicular to the luteal phase; SMD > 0 indicates an increase.

Potential publication bias was assessed by visually inspecting a funnel plot of all 47 effect sizes (SMDs) of CVA change from the follicular to the luteal phase derived from the 37 studies included in the meta-analysis described above (Figure 3). The effect sizes (SMDs) are plotted against the standard error of SMD with asymmetry in the plot providing a visual cue for potential bias. The symmetry of the funnel plot in Figure 3 seems somewhat limited, with slightly more outlying negative SMDs (indicating a luteal decrease in CVA) than outlying positive SMDs (indicating a luteal increase in CVA). However, since visual interpretation of funnel plots is considered inherently subjective [48], we additionally conducted an Egger test of small-study effects [92,93]. The test relies on the assumption that precision of the estimated SMD increases as the size of the study increases and examines whether the association between estimated SMD and study size (indicated by the standard error of SMD) is greater than can be expected to occur by chance [48]. The estimated bias coefficient is −1.2 with a standard error of 1.3, giving a *p*-value of 0.338. Under the null hypothesis of no small-study effects, the test, thus, provides no evidence for the presence of a small-study effect.

A total of seven publications (*n*_individuals_ = 107) did not report sufficient quantitative data in the manuscript and data could not be retrieved from the corresponding authors after four contact attempts [49,51,60,69,79,81,83]. These studies could not be included in the meta-analysis; however, their general results regarding the comparison between follicular and luteal cycle half are described here: One of the seven studies is consistent with the result of the meta-analysis, as they report a significant decrease in CVA from the follicular to the luteal phase [60]. Seebauer et al. [83] found a significant CVA decrease from the follicular to the luteal phase, but only in their subsample of females with a high mean HR. In contrast to the results of the meta-analysis below, individuals with a low mean HR showed an increase in CVA from the follicular to the luteal phase, suggesting that interindividual factors might have a moderating influence on the relationship between the menstrual cycle and CVA. Rawal and Saini [79] and Sato and Miyake [81] also found an increase in CVA from the follicular to the luteal phase in their samples; however, in both publications, the authors did not report any inferential statistics regarding this specific comparison. Three of the seven studies [49,51,69] reported no significant differences between their follicular and luteal CVA measures.

### 3.3. CVA Change in Finer-Grained Cycle Phase Comparisons

The broad division of the menstrual cycle into the follicular and luteal phases does not sufficiently capture the host of hormone changes and levels that occur across the menstrual cycle; therefore, we also examined comparisons between the five more specific cycle phases coded above (i.e., menstrual, mid-to-late follicular, ovulatory, early-to-mid luteal, and premenstrual phase). However, as mentioned before, meta-analytically comparing cycle phases that had been determined imprecisely by the authors of the original publications makes results less meaningful. For this reason, the finer-grained cycle phase comparisons were carried out solely on the basis of cycle phases identified as having been delineated rigorously (i.e., in accordance with the requirements for precise cycle phase determination outlined above).

All 38 studies (*n*_individuals_ = 1.018) were evaluated to determine whether each phase was determined precisely as described above. In summary, all 27 menstrual, and 20 out of the 23 mid-to-late follicular cycle phase observations could be included into the finer-grained phase comparisons. Three mid-to-late follicular observations had to be excluded since the authors of the original publications either did not specify on which cycle day(s) the CVA assessment took place [45,68] or because the CVA assessment in the original publication fell in equal parts into the mid-to-late follicular and ovulatory phase defined in the present paper [50]. Of the seven ovulatory CVA assessments, three had to be excluded since ovulation was solely determined using the forward-count method, which carries a high risk of error for ovulatory phase determination due to variability in follicular phase length [43,52,86]. Regarding the early-to-mid luteal phase, 20 out of the 24 CVA assessments in this phase had to be excluded since they did not meet our requirements for precise cycle phase determination: In 13 cases, the early-to-mid luteal cycle phase was merely determined by the forward-count method [50,52,53,58,62,64,71,72,75,78,86,89,90]. In two cases, early-to-mid luteal phase CVA assessments had to be excluded because the authors of the original publications did not specify their approach for determining the early-to-mid luteal cycle phase [45,68]. In another four cases, the period in which CVA assessments took place fell in equal parts in the early-to-mid luteal and premenstrual week [76,77,84,87]. Out of the 17 premenstrual CVA assessments, five had to be excluded from the finer-grained phase comparisons because the premenstrual cycle phase was determined exclusively using the forward-count approach which does not do different cycle lengths justice [43,50,54,67,88].

Out of the 38 studies which reported quantitative data suitable for meta-analytic calculations, 21 studies (*n*_individuals_ = 662) allowed for finer-grained cycle phase comparisons since they determined at least two of the five menstrual cycle phases according to our requirements (detailed above) for precise cycle determination [40,42,44,54,57,59,62,63,64,65,66,70,71,72,73,74,80,82,84,85,87]. Out of those 21 studies, 10 studies (*n*_individuals_ = 349) could be used for the phase comparison from menstrual CVA to the mid-to-late follicular CVA [40,44,54,62,63,64,72,84,85,87]. The comparison from the menstrual to ovulatory phase was carried out based on four studies [57,71,80,85] and a total of 47 females. Four studies (*n*_individuals_ = 86) allowed for a menstrual to early-to-mid luteal phase comparison [40,74,80,85]. Menstrual to premenstrual CVA was compared based on five studies [44,59,63,73,85] and a total of 200 females. The mid-to-late follicular and ovulatory phase and the ovulatory and premenstrual phase could not be meta-analytically compared since both comparisons were only reported by Tenan et al. [85]. Three studies (*n*_individuals_ = 53) allowed for the comparison of mid-to-late follicuar to early-to-mid luteal CVA [40,42,85], and eight studies (*n*_individuals_ = 280), the comparison of mid-to-late follicular to premenstrual CVA [42,44,63,65,66,70,82,85]. The comparison of the ovulatory and early-to-mid luteal cycle phase was based on two studies [80,85] and a total of 23 females. The studies by de Zambotti et al. [42] and Tenan et al. [85] with a total of 39 females were used for the early-to-mid luteal to premenstrual phase comparison. All corresponding datasets are provided in the Supplemental Materials.

The results of all eight meta-analytic cycle phase comparisons are displayed in Table 4. All but two cycle phase comparisons did not reach statistical significance (i.e., their 95% confidence interval intersected 0). Only the comparisons from the menstrual to the premenstrual phase (*d* = −1.17, 95% CI (−2.18, −0.17)) and from the mid-to-late follicular phase to the premenstrual phase (*d* = −1.32, 95% CI (−2.35, −0.29)) showed a significant decrease of CVA across the cycle with conventionally large effect sizes [91]. These phase comparisons will be displayed in more detail below.

#### 3.3.1. CVA Change from the Mid-To-Late Follicular to the Premenstrual Phase

**Figure 4:**
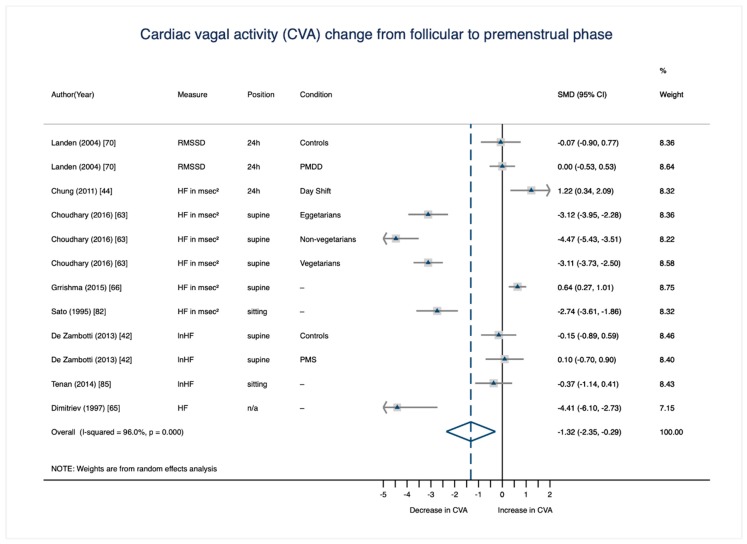
Meta-analysis on within-person change of CVA from the mid-to-late follicular to the premenstrual cycle phase (*n*_studies_ = 8; *n*_SMD_ = 12; *n*_individuals_ = 280). RMSSD = root mean square of successive differences between adjacent RR intervals; HF = high frequency component in the power spectrum range; n/a = information not available; PMDD = premenstrual dysphoric disorder; PMS = premenstrual syndrome.

The significant, large decrease of CVA from the mid-to-late follicular to the premenstrual phase (Figure 4) was based on data from 280 females across eight studies [42,44,63,65,66,70,82,85]. The study by Choudhary et al. [63] accounts for one fourth of the weight due to its large sample size. Study or subsample specific effect sizes of the CVA change from the mid-to-late follicular to the premenstrual phase vary from *d* = −4.41 to *d* = 1.22. Only two studies [44,66] and one subsample (the PMS group in the study by de Zambotti et al. [42]) show a contradictory CVA change across the cycle; that is, an increase from the mid-to-late follicular phase to the premenstrual week.

Two studies in this phase comparison compare healthy controls with females with premenstrual dysphoric disorder (PMDD [70]) and with females with premenstrual syndrome (PMS [42]). Since PMDD and PMS, as per their definitions, are associated with affective, cognitive, behavioral, and/or physical symptoms in the premenstrual week due to an abnormal response to normal hormonal changes [94], the decrease in CVA from the follicular phase to the premenstrual phase could be driven by these clinical subsamples. In order to examine this possibility, we repeated the mid-to-late follicular versus premenstrual cycle phase comparison without the PMDD subsample (*n* = 28) reported by Landén et al. [70] and without the PMS subsample (*n* = 12) reported by de Zambotti et al. [42]. Interestingly, the effect size of the decrease from the mid-to-late follicular to the premenstrual phase increased without these 40 PMS/PMDD participants, to *d* = −1.61, CI 95% (−2.88, −0.35). A meta-analytic comparison of the mid-to-late follicular phase and the premenstrual phase based on the 40 PMS/PMDD participants reported by Landén et al. [70] and de Zambotti et al. [42] revealed no significant change of CVA (*d* = 0.03, CI 95% (−0.42, 0.47)). As mentioned above, psychopathology is generally associated with reduced CVA [95,96]. More work is needed to understand whether individuals with PMS/PMDD demonstrate an abnormal or absent effect of normal ovarian hormone changes on CVA.

#### 3.3.2. CVA Change from the Menstrual to the Premenstrual Phase

**Figure 5:**
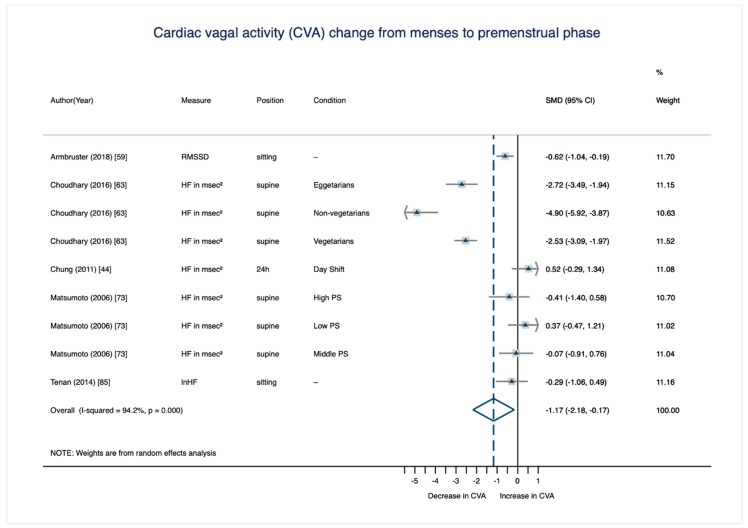
Meta-analysis on within-person change of CVA from the menstrual to the premenstrual cycle phase (*n*_studies_ = 5; *n*_SMD_ = 9; *n*_individuals_ = 200). RMSSD = root mean square of successive differences between adjacent RR intervals; HF = high frequency component in the power spectrum range; PS = premenstrual symptomatology.

The significant and strong decrease of CVA from the menstrual to the premenstrual phase (Figure 5) is based on data from 200 females across five studies [44,59,63,73,85]. However, two studies [63,73] each account for one third of the weight due to their large sample sizes. Study or subsample-specific effect sizes of the CVA-change from the menstrual to the premenstrual phase vary from *d* = −4.90 to *d* = 0.52. Only one study [44] and one subsample (the “low premenstrual symptomatology” group in the study by Matsumoto et al. [73]) reveal a contradictory CVA pattern across the cycle; that is, an increase from the menstrual to the premenstrual phase.

As above, this phase comparison was also repeated without the subgroup of participants reporting high premenstrual symptomatology (*n*_individuals_ = 8; [73]). The effect size of the decrease from the menstrual to the premenstrual phase consequently increased to *d* = −1.26, CI 95% (−2.36, −0.17). In line with this, taking only the subgroup of participants with high premenstrual symptomatology into account, analyses revealed a nonsignificant decrease of CVA from the menstrual to the premenstrual cycle phase of *d* = −0.41, CI 95% (−1.40, 0.58).

## 4. Discussion

This is the first systematic review and meta-analysis on the association between the menstrual cycle and cardiac vagal activity (CVA) with the aim of resolving the inconsistent findings in this literature and giving recommendations for future research. Based on a total of 45 studies included in the systematic review and a total of 38 studies included in various meta-analytic comparisons, we found compelling evidence for medium to large effects in differences between cycle phases, most broadly a significant decrease in CVA from the follicular to the luteal phase.

Inconsistencies in the definition of and the approach to determining cycle phases across the studies we included made reclassification of the phases necessary. In the process of reclassification, we developed systematic requirements for precise cycle-phase determination based on a variety of indices. We encourage future researchers to use these requirements when designing studies or meta-analyses to test hypotheses associated with specific cycle phases. We were able to recode a total of 21 (of 45) studies to determine precise fine-grained cycle phases (menstrual, mid-to-late follicular, ovulatory, midluteal, and premenstrual) and conducted pairwise, meta-analytic comparisons of each of these cycle phases. Statistically significant and physiologically relevant decreases in CVA were observed from the menstrual to the premenstrual and from the mid-to-late follicular to the premenstrual phase, both of which had a conventionally large effect size.

### 4.1. Potential Moderators and Mediators

Previous inconsistencies in the literature regarding the association between the menstrual cycle and CVA might be partially traced back to variables mediating or moderating this association. Nine out of the 45 studies included in this systematic review subsampled their participants as a function of interindividual differences: Two studies focused on predominantly physiological factors: Seebauer et al. [83] showed that females with a high average HR displayed a decrease from the follicular to the luteal phase, while females with a low average HR demonstrated increasing CVA levels from the follicular to the luteal measurement. Nakamura et al. [75] revealed a moderating effect of athletic state on cycle-dependent changes in CVA: while both the athletic and the control group showed decreasing levels from the follicular to luteal phase, the decrease in the athletic group was larger. Additionally, lifestyle factors like different diets take on a moderating role in the association between the menstrual cycle and CVA, since non-vegetarians displayed a larger decrease from follicular to luteal CVA levels than eggetarians and vegetarians [63]. The biggest set of studies investigating moderators and mediators addressed psychological factors: Liu et al. [72] showed changes of CVA levels in both the high and low neuroticism group, with neuroticism moderating the direction of change: while the highly neurotic group displayed a decrease in CVA levels from the follicular to the luteal phase, the less neurotic group demonstrated an increase. A total of five studies investigated differently severe levels of premenstrual symptomatology as potential mediators and moderators of cycle-dependent CVA fluctuations with varying results: two studies reported a decrease in CVA levels from the follicular to the luteal phase in both the control and PMS group; however, in one study this decrease was relatively larger in the PMS than in the control groups [42], while in the other study, the decrease was larger in the control group than in the PMS group [53]. In the study by Baker et al. [61], the control groups displayed no changes in CVA levels across the cycle, while the PMS group demonstrated a decrease from the follicular to the luteal phase. Matsumoto et al. [73] showed that the severity of premenstrual symptomatology (PS) moderated the direction of change in CVA levels: While the high PS group displayed a decrease, the low PS group demonstrated an increase in CVA levels from the follicular to the luteal assessment. Landén et al. [70] found no cycle-dependent CVA changes, neither in the PMDD group, nor in the control group.

It is known from the literature on PMS and PMDD that females differ in neurobiological and behavioral responses to menstrual cycle-related fluctuations in ovarian hormones, which is why some (and not all) females experience affective, cognitive, behavioral, and/or physiological symptoms in the premenstrual weeks [94]. In the context of PMS and PMDD, this hormone sensitivity has been linked to a variety of genetic factors [97,98], as well as environmental factors, including traumatic experiences [99,100,101] and recent life stress [102,103]. The database of this meta-analysis did not allow us to examine whether there is a unique effect of the cycle on CVA among individuals with PMS/PMDD, particularly given that these premenstrual disorders were not diagnosed in a standardized manner in the studies reviewed (see [104]). However, it is possible that changes in CVA across the cycle serve as biomarkers for neurobiological hormone sensitivity in some women with PMS/PMDD. This is an important topic for future female health research, and may provide insights regarding the pathophysiology and treatment of premenstrual disorders.

### 4.2. Underlying Mechanisms

In all of the studies included in this systematic review and meta-analysis measured, cycle phase was a proxy for changing E2 and P4 levels across the cycle (as opposed to actual E2 and P4 levels). While this focus on cycle phase is a reasonable initial approach, it allows only cautious interpretations regarding potential mechanisms underlying cyclical CVA fluctuations. For example, as outlined above, we defined the premenstrual week (which is associated with particularly low levels of CVA compared to the menstrual and mid-follicular phase) as the full seven days preceding the onset of menses. Since the premenstrual week is characterized by varying hormone levels—with E2 and P4 stable and elevated in the first half of the week and falling rapidly in the second half of the week—one cannot clearly attribute premenstrual week changes in CVA to any one hormonal event. However, prior work does suggest a few plausible underlying mechanisms, discussed below.

Previous research ascribes E2 and P4 profound effects and multiple mechanisms of action on mood, memory, mental state, neurodevelopmental, and neurodegenerative processes in mammals. For example, experimental animal studies suggest that the ovarian hormones E2 and P4 influence central-nervous regions through nongenomic and genomic effects [105] that co-localize with regions that are also important for efferent vagal control of the heart, described in the neurovisceral integration theory [11]. For example, higher levels of vagally-mediated CVA in the mid-to-late follicular phase might represent dopamine-enhancing effects of elevated E2 in the prefrontal cortex [38]. Since P4 rapidly metabolizes into calming GABAergic neuroactive steroid metabolites, such as allopregnanolone, the abrupt premenstrual withdrawal from P4 may cause a state of GABAergic withdrawal in critical brain regions for the maintenance of CVA (e.g., the paraventricular nucleus; [106]). This may reduce vagal activity at the sinoatrial node of the heart with positive chronotropic effects (i.e., increase in heart rate and decrease in variability), resulting in lower CVA premenstrually. Rapid premenstrual withdrawal from E2 may also play a role in reduced premenstrual CVA, given the evidence that circulating E2 promotes choline uptake and acetylcholine synthesis (ACh; the primary vagal neurotransmitter [96]) increasing vagally-mediated cardiac autonomic activity.

Animal research suggests a role for choline acetyltransferase (ChAT) in the regulation of the circadian rhythm of the cholinergic system [107,108]. In particular, it has been suggested that there is a sexual dimorphism such that in the non-neuronal cholinergic cardiac system in murine-model female hearts expressed ChAT more than male hearts [108]. Indeed an earlier study from our group found ChAT to be associated with HRV in humans [109]. However, a more recent large genome wide association study did not replicate the ChAT finding and found no sex differences in the genes associated with HRV [110]. Thus, the role of ChAT in humans, and specifically, its role in the circadian variation of HRV in females remains unclear.

Future observational studies with repeated measures of CVA across the menstrual cycle should additionally measure hormone levels to shed light on the ovarian hormone levels and changes that are most strongly correlated with varying CVA levels. Eventually, experimental work in animals and humans in which hormone levels and changes are directly manipulated will be required to delineate the causal effects of E2 and P4 on CVA.

### 4.3. Limitations

While our systematic review and meta-analytic analyses provide solid evidence for menstrual cycle-related change in CVA, some limitations are noteworthy: First, we were unable to include several covariates that should be considered in future studies. For example, as previous literature has shown, physical activity [111,112] is related to CVA, as well as menstrual irregularities [113]. The same goes for mental stress, which has been associated with both blunted CVA [32] and sensitivity to menstrual cycle-related hormone transitions [102,103]. However, since the vast majority of included studies did not report indices of body composition (e.g., body mass index), status of physical activity, and current stress level, they could not be controlled for in the present analyses. Second, the results of the present systematic review and meta-analysis can only be generalized to naturally-cycling females with regular menstrual cycles. It is not possible to draw conclusions for individuals who report menstrual irregularities and/or those who use hormonal contraception. Finally, the quality of a systematic review and meta-analysis depends to a large extent on the quality of the studies on which they are based. Yet, the formal test for (adverse) small studies effects (i.e., Egger test) did not indicate a publication bias. Additionally, by solely including longitudinal studies which allowed for within-person comparisons across the menstrual cycle, we avoided cross-sectional studies which are less meaningful due to their not taking inter-individual differences into account. However, as has already been shown in detail above, not all of those studies conducted a precise cycle phase determination, and therefore, many had to be excluded from finer-grained, meta-analytic phase comparisons. As a result, some of these comparisons had to be carried out on a very limited number of studies (and thus, females) which reduced statistical power. In two cases (i.e., the mid-to-late follicular versus ovulatory phase comparison and the ovulatory versus premenstrual phase comparison), no meta-analytic analyses were possible, since only one study comparing these two cycle phases had determined them precisely.

## 5. Conclusions and Recommendations

The present investigation reveals compelling evidence for physiologically relevant changes in cardiac vagal activity across the menstrual cycle; most broadly, a significant decrease in CVA from the follicular to the luteal menstrual cycle phase. The inconsistent results reported in the literature appear to have been partially resolved by applying a common definition of menstrual cycle phases. Based on this expert reclassification, requirements for a precise determination of cycle phases are presented. A failure to carefully measure the cycle phase of female subjects in CVA studies could greatly reduce the validity and significance of this work. However, given the well-documented negative consequences of excluding females from both animal and human research on the basis of expected cyclical hormone fluctuations, we strongly advise against the practice of excluding fertile females from CVA studies. Instead, we suggest females be included and that forward and backward-count cycle information be collected in order to make reasonably precise determination of menstrual cycle phase for use as a covariate (or a factor of central interest; see [114]). If a researcher simply wants to avoid this variability altogether in CVA studies with fertile female participants, we suggest that CVA measurements be performed in the mid-follicular phase. Using careful measurement and statistical covariates, researchers in this area can account for these conventionally large effects of the cycle on CVA, which may vastly increase their ability to observe various effects of interest. In addition to these methodological concerns, future studies should address the mechanisms underlying the menstrual cycle-related CVA fluctuations, as well as possible interindividual differences in the direction and magnitude of these fluctuations. Future research is also required to determine if and how these day-to-day changes in CVA across the menstrual cycle are associated with mental and physical health risks in naturally-cycling women of fertile age.

## Figures and Tables

**Table 1:** Overview of cardiac vagal activity (CVA) measures included in the systematic review and meta-analysis.

CVA Measure	Definition
RMSSD	Root mean square of successive differences between adjacent R–R intervals in milliseconds or log-transformed values
HF	High frequency component in the power spectrum range between 0.15 and 0.4 Hz in milliseconds squared, seconds squared, normalized units, or log-transformed values to the basis of 10 (log) or e (ln)
RSA	Respiratory sinus arrhythmia, defined as heart rate variability (HRV) in synchrony with respiration, by which the R–R interval is shortened during inspiration and prolonged during expiration [49].
BS	Baroreflex slope, defined as the resulting changes in R–R intervals from modulated carotid baroreceptor (sequential neck pressure and suction; [50]).
CBS	Cardiovagal baroreflex sensitivity, defined as the slope relating R–R interval and systolic blood pressure [51].
CVI	Cardiac vagal index, defined as the ratio of the longest and shortest R–R interval during a 4 s exercise test [52].
HRR	Heart rate (HR) reactivity, defined as the difference between peak HR during 3 min exercise and HR at the first minute post exercise (i.e., recovery; [40]).
DBT	Deep breathing test, defined as the difference between the longest and shortest R–R interval while breathing at resonance frequency (6 breath/min; inhale and exhale phase lasting each for 5 s). This can be also described as expiration to inspiration ratio at resonance frequency [53].
Valsalva	Valsalva ratio, defined as the ratio of the maximum R–R interval after strain and the shortest R–R interval during strain. Under the Valsalva maneuver, participants’ nostrils were closed by nose clip and participants were asked to blow by doing forceful expiration into the rubber tube of a mercury sphygmomanometer, raise the mercury column to 40 mm Hg and maintain that level for at least 15 s. HR was recorded continuously during the whole procedure [54].

**Table 2:** Overview of approaches to determine cycle phases.

Menstrual Cycle		Cycle-Day-Based Phase Determination		Ovulation-Based Phase Determination	
Menstrual cycle half	Menstrual cycle phase	Forward-count method in cycle days (assuming a 28-day cycle)	Backward-count method in cycle days	LH testing	Basal body temperature
Follicular	Menstrual	Day 1 to 7			
	Mid-to-late follicular	Day 8 to 12			
	Ovulatory	Day 13 to 16	Day –15 to –12	Day of and day +1 following positive LH test	Nadir just before temperature rise in the luteal phase and day +1
Luteal	Early-to-mid luteal	Day 17 to 21	Day −11 to −8	Day +2 to +7 following positive LH test	Day +2 to +7 following nadir
	Premenstrual	Day 22 to 28	Day −7 to −1	Day +8 to +14 following positive LH test	Day +8 to +14 following nadir

Note. LH = luteinizing hormone.

**Table 3:** Study characteristics of all 45 publications included in the systematic review and meta-analysis.

Names of the Authors (Year of Publication)	Sample Size	Potential Sub-Sampling of Sample of Interest (Between-Person)	Experi-mental Condition (Within-Person)	Cycle Phases Compared					Potential Validation Of Cycle Phase	Measure Indexing CVA	Body Position During CVA Assessment
				Menstrual	Mid-To-Late Follicular	Ovulatory	Early-To-Mid Luteal	Premenstrual			
Abidi et al. (2017) [58]	12	-	-	“Low hormone”: Day 2–5			“High hormone”: Day 18–24			HFnu	Supine
Armbruster et al. (2018) [59]	45	-	-	“Early follicular”: Day 1–7				“Late luteal”: Day 6–1 before menstrual onset	Saliva hormone analyses of E2, P4, Testosterone	RMSSD	Sitting
Bai et al. (2009) [60]	16	-	-		“Follicular”: Day = 11.9 ± 1.4; range 10–14		“Luteal”: Day = 22.0 ± 1.4; range 20–24		Blood hormone analyses of E2 and P4	HF in ms^2^	Supine
Baker et al. (2008) [61]	21	PMS versus Controls	-		“Mid-follicular” Day 6–12			“Late-luteal” Day 9–13 after LH-surge	Blood hormone analyses of P4	RMSSD	Supine
Balayssac-Siransy et al. (2014) [40]	14	-	Active Recovery 3rd min	“Menstrual”: Day = 2.9 ± 0.6; range 1–5	“Follicular”: Day = 13.0 ± 1.4; 4 days preceding ovulation		“Luteal”: Day = 23.1 “Luteal” 1.4; 6–10 days after ovulation		Blood hormone analyses of E2 and P4	HRR	Sitting
Brar et al. (2015) [62]	50	-	-	“Menstrual”: Day = 2	“Proliferative”: Day = 10		“Secretory”: Day = 21			RMSSD	Sitting
Choudhary et al. (2016) [63]	100	Eggetarians versus Non-vegetarians versus Vegetarians	-	“Menses”: Day 1–2	“Follicular”: Day 7–10			“Luteal”: Day 7–3 days before menstrual onset		HF in ms^2^	Supine
Chung & Yang (2011) [44]	12	-	Day Shift	“Menses”: Day 1–2	“Follicular”: Day 7–10			“Luteal”: Day 7–3 before menstrual onset	Blood hormone analyses of E2 and P4	HF in ms^2^	24 h
Cooke et al. (2002) [50]	10	-	-	Day 0–8	Day 9–14		Day 15–20	Day 21–25	Blood hormone analyses of E2 and P4	BS	Supine
De Zambotti et al. (2013) [42]	26	PMS versus Controls	REM sleep		“Mid-follicular”: Day 6–11		“Mid-luteal”: Day 5–9 after LH-surge on average 8 ± 2 days before menstrual onset	“Late-luteal”: Day 10–14 after LH-surge on average 3 ± 2 days before menstrual onset	Blood hormone analyses of E2 and P4	lnHF	Supine
Devaki et al. (2014) [64]	15	-	-	“Menstrual”: Day 1–5	“Proliferative”: Day 9–12		“Secretory”: Day 19–22			HFnu	Supine
Dimitriev et al. (2007) [65]	10	-	-		“Follicular”: Day 7–11			“Luteal”: Day 7–3 before menstrual onset		HF in ms^2^	n/a
Grrishma et al. (2015) [66]	60	-	-		“Follicular”: Day 8–10			“Luteal”: Day 5–1 before menstrual onset		HF in ms^2^	Supine
Guasti et al. (1999) [67]	13	-	-	“Follicular” Day 4–6				“Luteal”: Day 20–26		HFnu	Supine
Huang et al. (2015) [68]	10	-	-		“Mid-follicular”: n/a		“Mid-luteal”: n/a		Blood hormone analyses of P4	HFnu	n/a
Konishi et al. (2008) [69]	12	-	-		“Postmenstrual”: Day 3–7 after cessation of bleeding			“Premenstrual”: Day 10–3 before menstrual onset		HF in ms^2^	Seated
Kulshreshtha et al. (2013) [53]	45	PMS versus Controls	-	“Follicular”: Day = 7			“Luteal”: Day = 21			DBT	n/a
Landén et al. (2004) [70]	39	PMDD versus Controls	-		“Follicular”: Day 6–10			“Luteal”: Day 5–0 before menstrual onset		RMSSD	24 h
Leicht et al. (2003) [71]	10	-	-	“Menses”: Day = 3.8 ± 0.5; range 1–5		“Ovulation”: Day = 15.8 ± 0.7; range 11–21	“Luteal”: Day = 22.1 ± 0.4; range 21–24		Blood hormone analyses of E2 and P4	HF in ms^2^	Supine
Liu et al. (2013) [72]	27	High versus Low Neuroticism	-	“Menstruation”: Day 1–4	“Ovulation”: Day 11–13		“Luteal”: Day 21–22			HFnu	n/a
Lüthi et al. (2008) [49]	10	-	-	“Follicular”: Day 3–6		“Mid-cycle”: Day of/day after LH-surge	“Luteal”: Day 6–8 after LH-surge		Blood hormone analyses of E2 and P4	RSA	Seated
Matsumoto et al. (2006) [73]	30	High versus Middle versus Low Premenstrual Symptomatology	-	“Follicular”: Day 1–5				“Late luteal”: Day 7–1 before menstrual onset	Urinary hormone analyses of E2 and P4	HF in ms^2^	Supine
McKinley et al. (2009) [74]	49	-	-	“Early-to-mid-follicular”: Day 4–10			“Mid-luteal”: Day 3 after LH-surge–5 days before menstrual onset			logRMSSD	24 h
Minson et al. (2000) [51]	9	-	-	“Early follicular”: Day 2–4				“Midluteal”: Day 8–10 after LH-surge	Blood hormone analyses of E2, P4, LH, follicle stimulating hormone	CBS	Supine
Nakagawa et al. (2006) [75]	11	-	-		“Follicular”: Day = 9.5 ± 0.5; range 7–12		“Luteal”: Day = 23.2 ± 0.8; range 18–26		Blood hormone analyses of E2 and P4	RMSSD	n/a
Nakamura et al. (2013) [76]	18	Athlete versus Control	-	“Early follicular”: Day 4–8			“Middle luteal”: Day 5–10 after ovulation			lnHF	Supine
Ohara et al. (2015) [45]	7	-	Eating Trial		“Follicular”: n/a		“Luteal”: n/a			HF in ms^2^	Supine
Pestana et al. (2018) [77]	19	-	-	“Menstrual”: Day 1–9				“Luteal”: Day 15-day before menstrual onset		HFnu	Supine
Princi et al. (2005) [78]	6	-	-	“Menstrual”: Day 3“Follicular”: Day 7			“Luteal”: Day 21		Blood hormone analyses of E2 and P4	HF in ms^2^	Sitting
Rawal & Saini (2014) [79]	20	-	-	“Menstrual”: n/a	“Follicular”: n/a		“Luteal”: n/a			RMSSD	Supine
Saeki et al. (1997) [80]	10	-	-	“Menstrual”: Day 1–3	“Follicular”: Period between menstrual and ovulatory phases	“Ovulatory”: Period of 4 days beginning 3 days prior to the day of ovuluation	“Luteal”: Period between ovulatory and premenstrual phases	“Premenstrual”: Days 7–1 before menstrual onset	Blood hormone analyses of E2 and P4	HF in sec^2^	Supine
Sato & Miyake (2004) [81]	14	-	-		“Follicular”: Day 7–11			“Luteal”: Days 7–3 before menstrual onset		HF in ms^2^	Sitting
Sato et al. (1995) [82]	20	-	-		“Follicular”: Day 7–10			“Luteal”: Days 7–3 before menstrual onset	Blood hormone analyses of P4	HF in ms^2^	Sitting
Seebauer et al. (2002) [83]	26	Low versus medium versus high average HR		“Menstruation”: Day 1–4	“Early follicular”: Remaining days between end of menses and mid-follicular phase “Mid-follicular”: 3 days preceding late follicular phase “Late follicular”: 3 days preceding ovulation phase	“Ovulation”: Positive test result ± 1 day (duration 3 days)	“Early luteal”: 4 days following ovulation phase “Mid-luteal”: 4 days following ovulation phase	“Late-luteal”: Remaining days between end of mid-luteal phase and onset of menses		logRSA	Supine
Shetty et al. (2010) [84]	54	-	-	“Menstrual”: Day 1–5	“Follicular”: Day 6–14		“Luteal”: Day 15–28 or menstrual onset			HF in ms^2^	Supine
Teixeira et al. (2015) [52]	13	-	-	“Early follicular”: Day = 3.6 ± 1.2; range 1–5		“Ovulatory”: Day = 14.3 ± 08; range 13–16	“Mid-luteal”: Day = 21.3 ± 0.8; range 20–24		Blood hormone analyses of E2 and P4	CVI	Supine
Tenan et al. (2014) [85]	13	-	-	“Early follicular”: Equally spaced with late follicular phase from menstrual onset until ovulatory phase	“Late follicular”: Equally spaced with early follicular phase from menstrual onset until ovulatory phase	“Ovulatory”: 3-day period	“Midluteal”: Equally spaced with late luteal phase from end of ovulatory phase until menstrual onset	“Late luteal”: Equally spaced with early luteal phase from end of ovulatory phase until menstrual onset		lnHF	Sitting
Tousignant-Laflamme & Marchand (2009) [86]	29	-	-	“Menstrual”: Day 1–3		“Ovulatory”: Day 12–14	“Luteal”: Day 19–23		Blood hormone analyses of E2 and P4	HF in ms^2^	Sitting
Usha Rani et al. (2013) [87]	50	-	-	“Menstrual”: Day 1–5	“Follicular”: Day 6–14			“Luteal”: Day 15–28		HFnu	n/a
Veldhuijzen von Zanten et al. (2009) [88]	12	-	-	“Follicular”: Day 4–9				“Luteal”: Day 19–25	Blood hormone analyses of E2 and P4	RMSSD	Supine
Verma et al. (2018) [54]	50	-	-	“Menstrual”: Day 1–5	“Follicular”: Day 9–12			“Luteal phase”: Day 19–25		Valsalva	Sitting
Voronova et al. (2015) [43]	21	-	Spring	“Early follicular”: Day 7		“Late follicular”: Day 13 “Ovulation”: Day 16		“Luteal”: Day 24		RMSSD	Supine
Weissman et al. (2009) [57]	14			“Early follicular”: Day 2–3		“Preovulatory”: Just before ovulation			Blood hormone analyses of E2	HFnu	Supine
Yazar (2016) [89]	30	-	-		“Late follicular”: Day 9–13		“Mid-luteal”: Day 19–23			RMSSD	n/a
Yildirir et al. (2001) [90]	43	-	-		“Follicular”: Day 10–12		“Luteal”: Day 19–23			HF in m/sn^2^	Supine

Note. PMDD = premenstrual dysphoric disorder; PMS = premenstrual syndrome; PS = premenstrual symptomatology; LH = luteinizing hormone; E2 = estradiol; P4 = progesterone; RMSSD = root mean square of successive differences between adjacent RR intervals; HF = high frequency component in the power spectrum range; HRR = heart rate reactivity; CVI = cardiac vagal index; DBT = deep breathing test; Valsalva = Valsalva ratio; BS = baroreflex slope; RSA = respiratory sinus arrhythmia; CBS = cardiovagal baroreflex sensitivity; n/a = information not available.

**Table 4:** Overview of meta-analytic results of the finer-grained phase comparisons.

To From	Mid-To-Late Follicular	Ovulatory	Early-To-Mid Luteal	Premenstrual
Menstrual	*d* = 0.23 95% CI (−0.08, 0.55) *n*_studies_ = 10; *n*_individuals_ = 349	*d* = 0.35 95% CI (−0.45, 1.16) *n*_studies_ = 4; *n*_individuals_ = 47	*d* = 0.28 95% CI (−0.52, 1.08) *n*_studie;_ = 4; *n*_individuals_ = 86	***d* = −1.17** 95% CI (−2.18, −0.17) *n*_studies_ = 5; *n*_individuals_ = 200
Mid-to-late follicular		*n*_studies_ = 1; *n*_individuals_ = 13	*d* = −0.14 95% CI (−0.60, 0.32) *n*_studies_ = 3; *n*_individuals_ = 53	***d* = −1.32** 95% CI (−2.35, −0.29) *n*_studies_ = 8; *n*_individuals_ = 280
Ovulatory			*d* = −0.06 95% CI (−0.64, 0.51) *n*_studies_ = 2; *n*_individuals_ = 23	*n*_studies_ = 1; *n*_individuals_ = 13
Early-to-mid luteal				*d* = 0.20 95% CI (−0.25, 0.64) *n*_studies_ = 2; *n*_individuals_ = 39

Note. Significant effects (due to 95% CI not intersecting 0) are bolded. Negative effect sizes indicate a decrease in CVA.

## Supplementary Materials

The following are available online at https://www.mdpi.com/2077-0383/8/11/1946/s1, Supplementary Materials 1: Syntax used in data analysis (text document); Supplementary Materials 2: Dataset (excel file).

## Appendix A

Search terms (period until 26 October 2018)
**PubMed**
  (“Menstrual Cycle”[Mesh] OR “Estradiol”[Mesh] OR “Progesterone”[Mesh] OR “Testosterone”[Mesh]) AND
  (“HRV”[tiab] OR “Heart Rate Variability”[tiab] OR “Heart Rate Variabilities”[tiab] OR “Heart Period Variability”[tiab] OR “Heart Period Variabilities”[tiab] OR “Cardiac Autonomic Activity”[tiab] OR “Cardiac Autonomic Control”[tiab] OR “Cardiac Electrophysiology”[tiab] OR “Cardiac Reactivity”[tiab] OR “Cardiac Vagal Control”[tiab] OR “Autonomic Nervous System Activity”[tiab] OR “Parasympathetic Nervous System Activity”[tiab] OR “Vagal Tone”[tiab] OR “Vagal Activity”[tiab] OR “Vagal Reactivity”[tiab] OR “Cardiac Electrophysiology”[Mesh] OR “Vagus Nerve/physiology”[Mesh]) AND
  (“Humans”[MeSH])
**WebOfScience**
  TS = (((“Menstrual Cycle”) OR (“Luteal Phase”) OR (“Follicular Phase”) OR (“Ovarian Hormones”) OR (“Gonadal Hormones”) OR (“Gonadal Sex Hormones”) OR (“Sex Hormones”) OR (“Sex Steroids”) OR(“Estrogen”) OR (“Estradiol”) OR (“Progesterone”) OR (“Testosterone”)) AND
  ((“HRV”) OR (“Heart Rate Variability”) OR (“Heart Rate Variabilities”) OR (“Heart Period Variability”) OR (“Heart Period Variabilities”) OR (“Cardiac Autonomic Activity”) OR (“Cardiac Autonomic Control”) OR (“Cardiac Electrophysiology”) OR (“Cardiac Reactivity”) OR (“Cardiac Vagal Control”) OR (“Autonomic Nervous System Activity”) OR (“Parasympathetic Nervous System Activity”) OR (“Vagal Tone”) OR (“Vagal Activity”) OR (“Vagal Reactivity”)))
  Delimiters: Publication Years Timespan = All Years. Databases = SCI-EXPANDED, SSCI, CPCI-S, CPCI-SSH, BKCI-S, BKCI-SSH, CCR-EXPANDED, IC. Search language = Auto.
**PsychInfo**
  (SU “Menstrual Cycle” OR SU “Luteal Phase” OR SU “Follicular Phase” OR “Ovarian Hormones” OR “Gonadal Hormones” OR “Gonadal Sex Hormones” OR “Sex Hormones” OR “Sex Steroids” OR “Estrogen” OR “Estradiol” OR “Progesterone” OR “Testosterone”) AND
  (SU “HRV” OR SU “Heart Rate Variability” OR SU “Heart Rate Variabilities” OR SU “Heart Period Variability” OR SU “Heart Period Variabilities” OR SU “Cardiac Autonomic Activity” OR SU “Cardiac Autonomic Control” OR SU “Cardiac Electrophysiology” OR SU “Cardiac Reactivity” OR SU “Cardiac Vagal Control” OR SU “Autonomic Nervous System Activity” OR SU “Parasympathetic Nervous System Activity” OR SU “Vagal Tone” OR SU “Vagal Activity” OR SU “Vagal Reactivity” OR SU “Vagus Nerve” OR SU “Parasympathetic Nervous System” OR SU “Sympathetic Nervous System”)
  Population Group: Human; Exclude MEDLINE records; Population Group: Human AND Apply equivalent subjects; Apply related words; Also search within the full text of the articles on 2016-07-22 07:20 AM”
**Cochrane**
  #1 (MeSH descriptor: [Menstrual Cycle] explode all trees); #2 (MeSH descriptor: [Estradiol] explode all trees); #3 (MeSH descriptor: Progesterone] explode all trees); #4 (MeSH descriptor: [Testosterone] explode all trees); #5 (“HRV” OR “Heart Rate Variability” OR “Heart Rate Variabilities” OR “Heart Period Variability” OR “Cardiac Autonomic Activity” OR “Cardiac Autonomic Control” OR “Cardiac Electrophysiology” OR “Cardiac Reactivity” OR “Cardiac Vagal Control” OR “Autonomic Nervous System Activity” OR “Parasympathetic Nervous System Activity” OR “Vagal Tone” OR “Vagal Activity” OR “Vagal Reactivity”: ti, ab, kw (Word variations have been searched)); #6 ((#1 or #2 or #3 oe #4) and #5)
**EMBASE**
  (‘Menstrual Cycle’/mj OR ‘Luteal Phase’/mj OR ‘Follicular Phase’/mj OR ‘Ovarian Hormones’ OR ‘Gonadal Hormones’/mj OR ‘Gonadal Sex Hormones’ OR ‘Sex Hormones’/mj OR ‘Sex Steroids’ OR ‘Estrogen’/mj OR ‘Estradiol’/mj OR ‘Progesterone’/mj OR ‘Testosterone’/mj) AND
  (‘HRV’ OR ‘Heart Rate Variability’/mj OR ‘Heart Rate Variabilities’ OR ‘Heart Period Variability’ OR ‘Heart Period Variabilities’ OR ‘Cardiac Autonomic Activity’ OR ‘Cardiac Autonomic Control’ OR ‘Cardiac Electrophysiology’/mj OR ‘Cardiac Reactivity’ OR ‘Cardiac Vagal Control’ OR ‘Autonomic Nervous System Activity’ OR ‘Parasympathetic Nervous System Activity’ OR ‘Vagal Tone’/mj OR ‘Vagal Activity’/mj OR ‘Vagal Reactivity’ OR ‘Vagus Nerve’/mj OR ‘Parasympathetic Nervous System’/mj OR ‘Sympathetic Nervous System’/mj) AND
  ([Article]/lim OR [Article In Press]/lim OR [Editorial]/lim OR [Erratum]/lim OR [Letter]/lim OR [Note]/lim OR [Review]/lim OR [Short Survey]/lim) AND
  ([Adolescent]/lim OR [Young Adult]/lim OR [Adult]/lim) AND
  [Embase]/lim

## References

[1] Beauchaine T.P., Thayer J.F. (2015). Heart rate variability as a transdiagnostic biomarker of psychopathology. Int. J. Psychophysiol..

[2] Balzarotti S., Biassoni F., Colombo B., Ciceri M.R. (2017). Cardiac vagal control as a marker of emotion regulation in healthy adults: A review. Biol. Psychol..

[3] Thayer J.F., Lane R.D. (2000). A model of neurovisceral integration in emotion regulation and dysregulation. J. Affect. Disord..

[4] Thayer J.F., Hansen A.L., Saus-Rose E., Johnsen B.H. (2009). Heart rate variability, prefrontal neural function, and cognitive performance: The neurovisceral integration perspective on self-regulation, adaptation, and health. Ann. Behav. Med..

[5] Geisler F.C., Kubiak T., Siewert K., Weber H. (2013). Cardiac vagal tone is associated with social engagement and self-regulation. Biol. Psychol..

[6] Chalmers J.A., Heathers J.A., Abbott M.J., Kemp A.H., Quintana D.S. (2016). Worry is associated with robust reductions in heart rate variability: A transdiagnostic study of anxiety psychopathology. BMC Psychol..

[7] Kemp A.H., Quintana D.S. (2013). The relationship between mental and physical health: Insights from the study of heart rate variability. Int. J. Psychophysiol..

[8] Koenig J., Kemp A.H., Beauchaine T.P., Thayer J.F., Kaess M. (2016). Depression and resting state heart rate variability in children and adolescents—A systematic review and meta-analysis. Clin. Psychol. Rev..

[9] Sgoifo A., Carnevali L., Pico Alfonso M.A., Amore M. (2015). Autonomic dysfunction and heart rate variability in depression. Stress.

[10] Jarczok M.N., Aguilar-Raab C., Koenig J., Kaess M., Borniger J.C., Nelson R.J., Hall M., Ditzen B., Thayer J.F., Fischer J.E. (2018). The heart’s rhythm‘n’blues: Sex differences in circadian variation patterns of vagal activity vary by depressive symptoms in predominantly healthy employees. Chronobiol. Int..

[11] Thayer J.F., Lane R.D. (2009). Claude Bernard and the heart–brain connection: Further elaboration of a model of neurovisceral integration. Neurosci. Biobehav. Rev..

[12] Pavlov V.A., Chavan S.S., Tracey K.J. (2018). Molecular and functional neuroscience in immunity. Ann. Rev. Immunol..

[13] Jarczok M.N., Koenig J., Li J., Mauss D., Hoffmann K., Schmidt B., Fischer J.E., Thayer J.F. (2016). The association of work stress and glycemic status is partially mediated by autonomic nervous system function: Cross-sectional results from the Mannheim Industrial cohort study (MICS). PLoS ONE.

[14] Schuster A.K., Fischer J.E., Thayer J.F., Mauss D., Jarczok M.N. (2016). Decreased heart rate variability correlates to increased cardiovascular risk. Int. J. Cardiol..

[15] Thayer J.F., Yamamoto S.S., Brosschot J.F. (2010). The relationship of autonomic imbalance, heart rate variability and cardiovascular disease risk factors. Int. J. Cardiol..

[16] Pavlov V.A., Tracey K.J. (2012). The vagus nerve and the inflammatory reflex-linking immunity and metabolism. Nat. Rev. Endocrinol..

[17] Levine J.D., Khasar S.G., Green P.G. (2006). Neurogenic inflammation and arthritis. Ann. N.Y. Acad. Sci..

[18] Van Rensburg D.C., Ker J.A., Grant C.C., Fletcher L. (2012). Autonomic impairment in rheumatoid arthritis. Int. J. Rheum. Dis..

[19] De Couck M., Caers R., Spiegel D., Gidron Y. (2018). The role of the vagus nerve in cancer prognosis: A systematic and a comprehensive review. J. Oncol..

[20] Koenig J., Thayer J.F. (2016). Sex differences in healthy human heart rate variability: A meta-analysis. Neurosci. Biobehav. Rev..

[21] Woody M.L., McGeary J.E., Gibb B.E. (2014). Brooding rumination and heart rate variability in women at high and low risk for depression: Group differences and moderation by COMT genotype. J. Abnorm. Psychol..

[22] Laederach-Hofmann K., Mussgay L., Ruddel H. (2000). Autonomic cardiovascular regulation in obesity. J. Endocrinol..

[23] Koenig J., Jarczok M.N., Fischer J.E., Thayer J.F. (2015). The association of (effective and ineffective) analgesic intake, pain interference and heart rate variability in a cross-sectional occupational sample. Pain Med..

[24] Romanowicz M., Schmidt J.E., Bostwick J.M., Mrazek D.A., Karpyak V.M. (2011). Changes in heart rate variability associated with acute alcohol consumption: Current knowledge and implications for practice and research. Alcohol Clin. Exp. Res..

[25] Koenig J., Jarczok M.N., Kuhn W., Morsch K., Schäfer A., Hillecke T.K., Thayer J.F. (2013). Impact of caffeine on heart rate variability: A systematic review. J. Caffeine Res..

[26] Koenig J., Menke B., Hillecke T.K., Thayer J.F., Jarczok M.N. (2015). Heart rate variability and cocaine: A systematic review of human studies. Arch. Neurosci..

[27] Koenig J., Jarczok M.N., Wasner M., Hillecke T.K., Thayer J.F. (2014). Heart rate variability and swimming. Sports Med..

[28] Jarczok M.N., Jarczok M., Mauss D., Koenig J., Li J., Herr R.M., Thayer J.F. (2013). Autonomic nervous system activity and workplace stressors—A systematic review. Neurosci. Biobehav. Rev..

[29] Bilan A., Witczak A., Palusiński R., Myśliński W., Hanzlik J. (2005). Circadian rhythm of spectral indices of heart rate variability in healthy subjects. J. Electrocardiol..

[30] Goldberger J.J., Le F.K., Lahiri M., Kannankeril P.J., Ng J., Kadish A.H. (2006). Assessment of parasympathetic reactivation after exercise. Am. J. Physiol Heart Circ. Physiol.

[31] Kaikkonen P., Nummela A., Rusko H. (2007). Heart rate variability dynamics during early recovery after different endurance exercises. Eur. J. Appl. Physiol..

[32] Castaldo R., Melillo P., Bracale U., Caserta M., Triassi M., Pecchia L. (2015). Acute mental stress assessment via short term HRV analysis in healthy adults: A systematic review with meta-analysis. Biomed. Signal Proces. Control.

[33] Mihm M., Gangooly S., Muttukrishna S. (2011). The normal menstrual cycle in women. Anim. Reprod. Sci..

[34] Lenton E.A., Landgren B.M., Sexton L. (1984). Normal variation in the length of the luteal phase of the menstrual cycle: Identification of the short luteal phase. BJOG Int. J. Obstet. Gynaecol..

[35] Sundström Poromaa I., Gingnell M. (2014). Menstrual cycle influence on cognitive function and emotion processing—From a reproductive perspective. Front. Neurosci..

[36] Brinton R.D., Thompson R.F., Foy M.R., Baudry M., Wang J., Finch C.E., Morgan T.E., Pike C.J., Mack W.J., Stanczyk F.Z. (2008). Progesterone receptors: Form and function in brain. Front. Neuroendocrinol..

[37] Gruber C.J., Tschugguel W., Schneeberger C., Huber J.C. (2002). Production and actions of estrogens. N. Engl. J. Med..

[38] Jacobs E., D’Esposito M. (2011). Estrogen shapes dopamine-dependent cognitive processes: Implications for women’s health. J. Neurosci..

[39] Moher D., Liberati A., Tetzlaff J., Altman D.G. (2009). Preferred reporting items for systematic reviews and meta-analyses: The PRISMA statement. Ann. Intern. Med..

[40] Balayssac-Siransy E., Ouattara S., Adoubi A., Kouame A., Sall F., Bogui R. (2014). Effects of menstrual cycle on vagal reactivation in post-exercise recovery among young black African women. Sci. Sport.

[41] Lahiri M.K., Kannankeril P.J., Goldberger J.J. (2008). Assessment of autonomic function in cardiovascular disease: Physiological basis and prognostic implications. J. Am. Coll. Cardiol..

[42] De Zambotti M., Nicholas C.L., Colrain I.M., Trinder J.A., Baker F.C. (2013). Autonomic regulation across phases of the menstrual cycle and sleep stages in women with premenstrual syndrome and healthy controls. Psychoneuroendocrinology.

[43] Voronova N., Meigal A.Y., Yelaeva L., Kuzmina G. (2015). Heart rate variability in women during various seasons and phases of the menstrual cycle. Ekologiia Cheloveka.

[44] Chung M.-H., Yang C.C. (2011). Heart rate variability across the menstrual cycle in shift work nurses. J. Exp. Clin. Med..

[45] Ohara K., Okita Y., Kouda K., Mase T., Miyawaki C., Nakamura H. (2015). Cardiovascular response to short-term fasting in menstrual phases in young women: An observational study. BMC Womens Health.

[46] Laborde S., Mosley E., Thayer J.F. (2017). Heart rate variability and cardiac vagal tone in psychophysiological research–recommendations for experiment planning, data analysis, and data reporting. Front. Psychol..

[47] Harris R.J., Deeks J.J., Altman D.G., Bradburn M.J., Harbord R.M., Sterne J.A. (2008). Metan: Fixed-and random-effects meta-analysis. Stata J..

[48] Harbord R.M., Harris R.J., Sterne J.A. (2009). Updated tests for small-study effects in meta-analyses. Stata J..

[49] Lüthi M., Roach D.E., Beaudin A.E., Debert C.T., Sheldon R.S., Poulin M.J. (2008). Effects of ovarian hormones and aging on respiratory sinus arrhythmia and breathing patterns in women. Clin. Auton. Res..

[50] Cooke W.H., Ludwig D.A., Eckberg D.L., Convertino V.A. (2002). Does the menstrual cycle influence the sensitivity of vagally mediated baroreflexes?. Clin. Sci..

[51] Minson C.T., Halliwill J.R., Young T.M., Joyner M.J. (2000). Influence of the menstrual cycle on sympathetic activity, baroreflex sensitivity, and vascular transduction in young women. Circulation.

[52] Teixeira A.L., Ramos P.S., Vianna L.C., Ricardo D.R. (2015). Effects of ovarian hormones and oral contraceptive pills on cardiac vagal withdrawal at the onset of dynamic exercise. PLoS ONE.

[53] Kulshreshtha M., Kumar Y., Agarwal V., Dhama V. (2013). Symathovagal imbalance in premenstrual syndrome. Ind. J. Physiol. Pharmacol..

[54] Verma S., Khuraiya P., Soni R. (2018). A comparative study of parasympathetic function tests during different phases of menstrual cycle in young healthy females. Int. J. Res. Med. Sci.

[55] Edler C., Lipson S.F., Keel P.K. (2007). Ovarian hormones and binge eating in bulimia nervosa. Psychol. Med..

[56] Eisenlohr-Moul T.A., Schmalenberger K.M., Owens S.A., Peters J.R., Dawson D.N., Girdler S.S. (2018). Perimenstrual exacerbation of symptoms in borderline personality disorder: Evidence from multilevel models and the Carolina Premenstrual Assessment Scoring System. Psychol. Med..

[57] Weissman A., Lowenstein L., Tal J., Ohel G., Calderon I., Lightman A. (2009). Modulation of heart rate variability by estrogen in young women undergoing induction of ovulation. Eur. J. Appl. Physiol..

[58] Abidi S., Nili M., Serna S., Kim S., Hazlett C., Edgell H. (2017). Influence of sex, menstrual cycle, and oral contraceptives on cerebrovascular resistance and cardiorespiratory function during Valsalva or standing. J. Appl. Physiol..

[59] Armbruster D., Grage T., Kirschbaum C., Strobel A. (2018). Processing emotions: Effects of menstrual cycle phase and premenstrual symptoms on the startle reflex, facial EMG and heart rate. Behav. Brain Res..

[60] Bai X., Li J., Zhou L., Li X. (2009). Influence of the menstrual cycle on nonlinear properties of heart rate variability in young women. Am. J. Physiol Heart Circ. Physiol.

[61] Baker F.C., Colrain I.M., Trinder J. (2008). Reduced parasympathetic activity during sleep in the symptomatic phase of severe premenstrual syndrome. J. Psychosom. Res..

[62] Brar T.K., Singh K.D., Kumar A. (2015). Effect of different phases of menstrual cycle on heart rate variability (HRV). J. Clin. Diagn. Res..

[63] Choudhary A.K., Alam T., Jiwane R., Kishanrao S.S. (2016). A comparative analysis of dietary habits on sensory motor association and heart rate variability during menstrual cycle. J. Clin. Diagn. Res..

[64] Devaki P., Saikumar P., Prabhu K., Prasannavenkatesh E., Kalaiselvi V. (2014). Heartrate variability across different phases menstrual cycle in adolescent females. Res. J. Pharm. Biol. Chem. Sci..

[65] Dimitriev D., Saperova E., Dimitriev A., Karpenko I. (2007). Features of cardiovascular functioning during different phases of the menstrual cycle. Rossiiskii Fiziologicheskii Zhurnal Imeni IM Sechenova.

[66] Grrishma B., Gaur G., Chaturvedula L., Velkumary S., Subramanian S.K., Gurunandan U. (2015). Assessment of cardiovascular autonomic functions and baroreceptor reactivity in women with premenstrual syndrome. Ind. J. Physiol. Pharmacol..

[67] Guasti L., Grimoldi P., Mainardi L.T., Petrozzino M.R., Piantanida E., Garganico D., Diolisi A., Zanotta D., Bertolini A., Ageno W. (1999). Autonomic function and baroreflex sensitivity during a normal ovulatory cycle in humans. Acta Cardiol..

[68] Huang S.-C., Wong A.M., Ho C.-W., Weng T.-P., Cheng S.-C., Wang J.-S. (2015). Comparison of cardiac autonomic nervous system disturbed by sleep deprivation in sex and menstrual phase. Chin. J. Physiol..

[69] Konishi K., Kumashiro M., Izumi H., Higuchi Y. (2008). Effects of the menstrual cycle on working memory: Comparison of postmenstrual and premenstrual phases. Ind. Health.

[70] Landén M., Wennerblom B., Tygesen H., Modigh K., Sörvik K., Ysander C., Ekman A., Nissbrandt H., Olsson M., Eriksson E. (2004). Heart rate variability in premenstrual dysphoric disorder. Psychoneuroendocrinology.

[71] Leicht A.S., Hirning D.A., Allen G.D. (2003). Heart rate variability and endogenous sex hormones during the menstrual cycle in young women. Exp. Physiol..

[72] Liu Q., Zhou R., Oei T.P., Wang Q., Zhao Y., Liu Y. (2013). Variation in the stress response between high-and low-neuroticism female undergraduates across the menstrual cycle. Stress.

[73] Matsumoto T., Ushiroyama T., Morimura M., Moritani T., Hayashi T., Suzuki T., Tatsumi N. (2006). Autonomic nervous system activity in the late luteal phase of eumenorrheic women with premenstrual symptomatology. J. Psychosom. Obst. Gyn..

[74] McKinley P.S., King A.R., Shapiro P.A., Slavov I., Fang Y., Chen I.S., Jamner L.D., Sloan R.P. (2009). The impact of menstrual cycle phase on cardiac autonomic regulation. Psychophysiology.

[75] Nakagawa M., Ooie T., Takahashi N., Taniguchi Y., Anan F., Yonemochi H., Saikawa T. (2006). Influence of menstrual cycle on QT interval dynamics. Pacing Clin. Electrophysiol..

[76] Nakamura M., Hayashi K., Aizawa K., Mesaki N., Kono I. (2013). Effects of regular aerobic exercise on post-exercise vagal reactivation in young female. Eur. J. Sport Sci..

[77] Pestana E.R., Mostarda C.T., Silva-Filho A.C., Salvador E.P., de Carvalho W.R.G. (2018). Effect of different phases of menstrual cycle in heart rate variability of physically active women. Sport Sci. Health.

[78] Princi T., Parco S., Radillo O., De Seta F., Ulcigrai L., Accardo A. (2005). Heart rate variability and menstrual cycle in eumenorrheic young women. Biomed. Sci. Instrum..

[79] Rawal K., Saini I. (2014). Comparative analysis of measuring heart rate variability during different phases of menstrual cycle in young healthy women. Int. J. Inf. Electron. Eng..

[80] Saeki Y., Atogami F., Takahashi K., Yoshizawa T. (1997). Reflex control of autonomic function induced by posture change during the menstrual cycle. J. Auton. Nerv. Syst..

[81] Sato N., Miyake S. (2004). Cardiovascular reactivity to mental stress: Relationship with menstrual cycle and gender. J. Physiol. Anthropol. Appl. Hum. Sci..

[82] Sato N., Miyake S., Akatsu J.I., Kumashiro M. (1995). Power spectral analysis of heart rate variability in healthy young women during the normal menstrual cycle. Psychosom. Med..

[83] Seebauer M., Frühwirth M., Moser M. (2002). Changes of respiratory sinus arrhythmia during the menstrual cycle depend on average heart rate. Eur. J. Appl. Physiol..

[84] Shetty S., Pai S.R., Nayanatara A., Bhat M.R., Shetty B.A. (2010). Comparative study of time and frequency domain analysis of heart rate variability in different phases of menstrual cycle. J. Chin. Clin. Med..

[85] Tenan M.S., Brothers R.M., Tweedell A.J., Hackney A.C., Griffin L. (2014). Changes in resting heart rate variability across the menstrual cycle. Psychophysiology.

[86] Tousignant-Laflamme Y., Marchand S. (2009). Autonomic reactivity to pain throughout the menstrual cycle in healthy women. Clin. Auton. Res..

[87] Usha Rani Y.S., Manjunath P., Desai R.D. (2013). Comparative study of heart rate variability, heart rate and blood pressure in different phases of menstrual cycle in healthy young women aged 18–22 years. J. Phys. Pharm. Adv..

[88] Veldhuijzen van Zanten J.J., Carroll D., Ring C. (2009). Mental stress-induced haemoconcentration in women: Effects of menstrual cycle phase. Br. J. Health Psychol..

[89] Yazar Ş. (2016). Impact of menstrual cycle on cardiac autonomic function assessed by heart rate variability and heart rate recovery. Med. Prin. Pract..

[90] Yildirir A., Kabakci G., Akgul E., Tokgozoglu L., Oto A. (2001). Effects of menstrual cycle on cardiac autonomic innervation as assessed by heart rate variability. Ann. Noninvasive Electrocardiol..

[91] Cohen J. (1970). Approximate power and sample size determination for common one-sample and two-sample hypothesis tests. Educ. Psychol. Meas..

[92] Egger M.G., Smith G.D., Schneider M., Minder C. (1997). Bias in meta-analysis detected by a simple graphical test. BMJ.

[93] Sterne J.A., Gavaghan D., Egger M. (2000). Publication and related bias in meta-analysis: Power of statistical tests and prevalence in the literature. J. Clin. Epidemiol..

[94] Schmidt P.J., Nieman L.K., Danaceau M.A., Adams L.F., Rubinow D.R. (1998). Differential behavioral effects of gonadal steroids in women with and in those without premenstrual syndrome. N. Engl. J. Med..

[95] Kemp A.H., Koenig J., Thayer J.F. (2017). From psychological moments to mortality: A multidisciplinary synthesis on heart rate variability spanning the continuum of time. Neurosci. Biobehav. Rev..

[96] Thayer J.F., Åhs F., Fredrikson M., Sollers J.J., Wager T.D. (2012). A meta-analysis of heart rate variability and neuroimaging studies: Implications for heart rate variability as a marker of stress and health. Neurosci. Biobehav. Rev..

[97] Dubey N., Hoffman J.F., Schuebel K., Yuan Q., Martinez P.E., Nieman L.K., Rubinow D.R., Schmidt P.J., Goldman D. (2017). The ESC/E (Z) complex, an effector of response to ovarian steroids, manifests an intrinsic difference in cells from women with premenstrual dysphoric disorder. Mol. Psychiatry.

[98] Huo L., Straub R.E., Roca C., Schmidt P.J., Shi K., Vakkalanka R., Weinberger D.R., Rubinow D.R. (2007). Risk for premenstrual dysphoric disorder is associated with genetic variation in ESR1, the estrogen receptor α gene. Biol. Psychiatry.

[99] Eisenlohr-Moul T.A., Rubinow D.R., Schiller C.E., Johnson J.L., Leserman J., Girdler S.S. (2016). Histories of abuse predict stronger within-person covariation of ovarian steroids and mood symptoms in women with menstrually related mood disorder. Psychoneuroendocrinology.

[100] Girdler S.S., Thompson K.S., Light K.C., Leserman J., Pedersen C.A., Prange A.J. (2004). Historical sexual abuse and current thyroid axis profiles in women with premenstrual dysphoric disorder. Psychosom. Med..

[101] Perkonigg A., Yonkers K.A., Pfister H., Lieb R., Wittchen H.-U. (2004). Risk factors for premenstrual dysphoric disorder in a community sample of young women: The role of traumatic events and posttraumatic stress disorder. J. Clin. Psychiatry.

[102] Gollenberg A.L., Hediger M.L., Mumford S.L., Whitcomb B.W., Hovey K.M., Wactawski-Wende J., Schisterman E.F. (2010). Perceived stress and severity of perimenstrual symptoms: The BioCycle Study. J. Womens Health.

[103] Jahromi B.N., Pakmehr S., Hagh-Shenas H. (2011). Work stress, premenstrual syndrome and dysphoric disorder: Are there any associations?. Iran. Red Crescent Med. J..

[104] Eisenlohr-Moul T.A., Girdler S.S., Schmalenberger K.M., Dawson D.N., Surana P., Johnson J.L., Rubinow D.R. (2017). Toward the reliable diagnosis of DSM-5 premenstrual dysphoric disorder: The Carolina Premenstrual Assessment Scoring System (C-PASS). Am. J. Psychiatry.

[105] Salerni S., Di Francescomarino S., Cadeddu C., Acquistapace F., Maffei S., Gallina S. (2015). The different role of sex hormones on female cardiovascular physiology and function: Not only oestrogens. Eur. J. Clin. Investig..

[106] Brunton P.J., Donadio M.V., Yao S.T., Greenwood M., Seckl J.R., Murphy D., Russell J.A. (2015). 5α-reduced neurosteroids sex-dependently reverse central prenatal programming of neuroendocrine stress responses in rats. J. Neurosci..

[107] Hut R.A., van der Zee E.A. (2011). The cholinergic system, circadian rhythmicity, and time memory. Behav. Brain Res..

[108] Oikawa S., Kai Y., Mano A., Ohata H., Nemoto T., Kakinuma Y. (2017). Various regulatory modes for circadian rhythmicity and sexual dimorphism in the non-neuronal cardiac cholinergic system. J. Cardiovas. Transl. Res..

[109] Wang X., Riese H., Su S., Zhu H., Dong Y., Ding X., Thayer J.F., Treiber F., Snieder H. (2009). A gene-wide association study of heart rate variability at rest and during stress: 8 genes in the parasympathetic pathway. Abstracts From the 2009 Joint Conference-Nutrition, Physical Activity and Metabolism and 49th Cardiovascular Disease Epidemiology and Prevention, Proceedings of the 2009 Joint Conference-Nutrition, Physical Activity and Metabolism and 49th Cardiovascular Disease Epidemiology and Prevention, Palm Harbor, FL, USA, 11–14 March 2009.

[110] Nolte I.M., Munoz M.L., Tragante V., Amare A.T., Jansen R., Vaez A., Von Der Heyde B., Avery C.L., Bis J.C., Dierckx B. (2017). Genetic loci associated with heart rate variability and their effects on cardiac disease risk. Nat. Commun..

[111] Koenig J., Jarczok M.N., Warth M., Ellis R., Bach C., Hillecke T., Thayer J.F. (2014). Body mass index is related to autonomic nervous system activity as measured by heart rate variability—A replication using short term measurements. J. Nutr. Health Aging.

[112] Koenig J., Windham B., Ferrucci L., Sonntag D., Fischer J., Thayer J.F., Jarczok M.N. (2015). Association strength of three adiposity measures with autonomic nervous system function in apparently healthy employees. J. Nutr. Health Aging.

[113] Russell J.B., Mitchell D., Musey P.I., Collins D.C. (1984). The relationship of exercise to anovulatory cycles in female athletes: Hormonal and physical characteristics. Obstet. Gynecol..

[114] Schmalenberger K.M., Eisenlohr-Moul T.A. (2019). Studying the menstrual cycle as an independent variable: Practical recommendations and tools for getting started. OSF Preprints.

