# Supplementary material for: A Systematic Review and Meta-Analysis of Within-Person Changes in Cardiac Vagal Activity across the Menstrual Cycle: Implications for Female Health and Future Studies
Source: J Clin Med. 2019 Nov 12;8(11):1946. doi: 10.3390/jcm8111946 (PMC6912442; doi:10.3390/jcm8111946)
Supplement: Supplementary file 1 [file jcm-08-01946-s001.zip › jcm-618911-supplementary/jcm-618911-Supplementary Materials 1.docx]

**Online Supplement:**

**Syntax Used in Data Analysis**

**Content:**

[1. Follicular vs. Luteal Phase Comparison 2](#_Toc23337196)

[2. Funnel Plot and Egger Test 3](#_Toc23337197)

[3. Menstrual vs. Mid-to-late Follicular Phase Comparison 3](#_Toc23337198)

[4. Menstrual vs. Ovulatory Phase Comparison 5](#_Toc23337199)

[5. Menstrual vs. Early-to-mid Luteal Phase Comparison 6](#_Toc23337200)

[6. Menstrual vs. Premenstrual Phase Comparison 7](#_Toc23337201)

[6.1 Menstrual vs. Premenstrual Phase Comparison – PMS/PMDD Subgroups Excluded 9](#_Toc23337202)

[7. Mid-to-late Follicular vs. Ovulatory Phase Comparison 11](#_Toc23337203)

[8. Mid-to-late Follicular vs. Early-to-mid Luteal Phase Comparison 11](#_Toc23337204)

[9. Mid-to-late Follicular vs. Premenstrual Phase Comparison 12](#_Toc23337205)

[9.1 Mid-to-late Follicular vs. Premenstrual Phase Comparison – PMS/PMDD Subgroups Excluded 14](#_Toc23337206)

[10. Ovulatory vs. Early-to-mid Luteal Phase Comparison 15](#_Toc23337207)

[11. Ovulatory vs. Premenstrual Phase Comparison 16](#_Toc23337208)

[12. Early-to-mid Luteal vs. Premenstrual Phase Comparison 17](#_Toc23337209)

[13. Ovulatory vs. Premenstrual Phase Comparison 18](#_Toc23337210)

# 1. Follicular vs. Luteal Phase Comparison

*change the current working directory to the specified drive and directory and import files

cd "[insert your path here]"

import excel "jcm-618911-Supplemental Materials 2.xlsx", sheet("1 Follicular vs. Luteal") firstrow clear case(lower)

destring _all , replace

*calculate SD from SE

replace sdfollikulär = sqrt(nfollikulär)*sdfollikulär if cavesevssdmeanvsmedian=="SE instead of SD"

replace sdluteal = sqrt(nluteal)*sdluteal if cavesevssdmeanvsmedian=="SE instead of SD"

*set order of HRV parameters in Graph

tab measure,m

gen order =.

replace order = 1 if measure=="RMSSD"

replace order = 2 if measure=="logRMSSD"

replace order = 3 if measure=="HF in msec²"

replace order = 4 if measure=="HF in sec²"

replace order = 5 if measure=="HF in m/sn²"

replace order = 6 if measure=="lnHF"

replace order = 7 if measure=="HFnu"

replace order = 8 if measure=="HF power (%)"

replace order = 9 if measure=="HF power in arbitrary units"

replace order = 10 if measure=="HRR"

replace order = 11 if measure=="HRR%"

replace order = 12 if measure=="CVI"

replace order = 13 if measure=="DBT"

replace order = 14 if measure=="Lying-Standing Ratio"

replace order = 15 if measure=="Operational Point (%)"

replace order = 16 if measure=="Valsalva"

replace order = 17 if measure=="BS (ms/mmHg)"

lab var firstauthor "Author(Year)"

lab var withinperson "Within Person"

lab var measure "Measure"

*drop if order==3

tab position

replace position = "-" if position==" not reported"

replace position = "-" if position==""

* calculate the Meta Analysis with specifies a random-effects model using the DerSimonian and Laird method,

* with the estimate of heterogeneity being taken from the inverse-variance fixed-effect model.

* useb ==1 means that the variable use (version b) must be on (selctetion in ecxeltable wich parameter to be reported

* e.g. if RMSSD and HFpower were available, useb inidcates the selected HRV-parameter

#delimit ;

metan nluteal mluteal sdluteal nfollikulär mfollikulär sdfollikulär if useb==1, randomi

xlabel( -6, -5, -4, -3, -2, -1 ,0 ,1, 2 ,3) force

xtick( -5.5, -4.5, -3.5, -2.5, -1.5, -0.5 ,0.5, 1.5, 2.5)

astext(75)

sortby(order firstauthor condition)

lcols(firstauthor measure position condition)

boxopt(mcolor(gs13))

diamopt(lcolor(navy))

pointopt( msymbol(T) mcolor(navy) msize(tiny))

ciopt( lcolor(gs8) lwidth(medium) )

olineopt(lcolor(navy) lpattern(dash))

favours("Decrease in CVA # Increase in CVA")

title("Cardiac vagal activity (CVA) change from follicular to luteal phase", size(small))

graphregion(fcolor(white) lcolor(gs15) ifcolor(white) ilcolor(white)) ;

#delimit cr

graph save "1 Follicular_Luteal_all_author.gph" , replace

graph export "1 Follicular_Luteal_all_author.png", replace

# 2. Funnel Plot and Egger Test

metafunnel _ES _seES if useb==1, egger

         ytitle(Standard Error of SMD )

         ylabel( 0(0.1)0.9, angle(horizontal))

         xtitle(SMD)

         xlabel(-4(1)2  )

         xmtick(-4.5(0.5)2.5 , nolabel)

          graphregion(fcolor(white) lcolor(gs15) ifcolor(white) ilcolor(white));

# 3. Menstrual vs. Mid-to-late Follicular Phase Comparison

*change the current working directory to the specified drive and directory and import files

cd "[insert your path here]"

import excel " jcm-618911-Supplemental Materials 2.xlsx", sheet("2 Menses vs. Follicular") firstrow clear case(lower)

destring _all , replace

d

tab position

replace position = "-" if position=="not reported"

replace position = "-" if position==""

*calculate SD from SE

replace sdmenses = sqrt(nmenses)*sdmenses if cavesevssdmeanvsmedian=="SE instead of SD"

replace sdfollicular = sqrt(nfollicular)*sdfollicular if cavesevssdmeanvsmedian=="SE instead of SD"

*set order of HRV parameters in Graph

tab measure,m

gen order =.

replace order = 1 if measure=="RMSSD"

replace order = 2 if measure=="HF"

replace order = 3 if measure=="lnHF"

replace order = 4 if measure=="HFnu"

replace order = 5 if measure=="HF in sec²"

replace order = 6 if measure=="HF m/sn²"

replace order = 7 if measure=="HF power (%)"

replace order = 8 if measure=="HF power in arbitrary units"

replace order = 8 if measure=="HRR"

replace order = 10 if measure=="HRR%"

replace order = 11 if measure=="CVI"

replace order = 12 if measure=="DBT"

replace order = 13 if measure=="Lying-Standing Ratio"

replace order = 14 if measure=="Operational Point (%)"

replace order = 15 if measure=="Valsalva"

replace order = 15 if measure=="Baroreflex Slope (ms/mmHg)"

gen str Info = measure + " " + position + " " + condition

* calculate the Meta Analysis with specifies a random-effects model using the DerSimonian and Laird method,

* with the estimate of heterogeneity being taken from the inverse-variance fixed-effect model.

* useb ==1 means that the variable use (version b) must be on (selctetion in ecxeltable wich parameter to be reported

* e.g. if RMSSD and HFpower were available, useb inidcates the selected HRV-parameter

#delimit ;

metan nfollicular mfollicular sdfollicular nmenses mmenses sdmenses if useb==1, randomi

xlabel( -2, -1 ,0 ,1, 2) force

xtick( -1.5, -0.5 ,0.5, 1.5)

sortby(order condition firstauthor)

astext(80)

textsize(110)

lcols(firstauthor measure position condition)

boxopt(mcolor(gs13))

diamopt(lcolor(navy))

pointopt( msymbol(T) mcolor(navy) msize(tiny))

ciopt( lcolor(gs8) lwidth(medium) )

olineopt(lcolor(navy) lpattern(dash))

favours("Decrease in VA # Increase in VA")

title("Vagal Activity (VA) Change from Menses to Follicular Phase", size(small))

graphregion(fcolor(white) lcolor(gs15) ifcolor(white) ilcolor(white)) ;

#delimit cr

graph save "2.1 Menses_Follicular.gph" , replace

graph export "2.1 Menses_Follicular.png", replace

# 4. Menstrual vs. Ovulatory Phase Comparison

*change the current working directory to the specified drive and directory and import files

cd "[insert your path here]"

import excel " jcm-618911-Supplemental Materials 2.xlsx", sheet("2 Menses vs. Ovulatory") firstrow clear case(lower)

destring _all , replace

d

***calculate SD from SE

*-*

*Not necessary for 2.2

*set order of HRV parameters in Graph

tab measure,m

gen order =.

replace order = 1 if measure=="RMSSD"

replace order = 2 if measure=="HF"

replace order = 3 if measure=="lnHF"

replace order = 4 if measure=="HFnu"

replace order = 5 if measure=="HF in sec²"

replace order = 6 if measure=="HF m/sn²"

replace order = 7 if measure=="HF power (%)"

replace order = 8 if measure=="HF power in arbitrary units"

replace order = 8 if measure=="HRR"

replace order = 10 if measure=="HRR%"

replace order = 11 if measure=="CVI"

replace order = 12 if measure=="DBT"

replace order = 13 if measure=="Lying-Standing Ratio"

replace order = 14 if measure=="Operational Point (%)"

replace order = 15 if measure=="Valsalva"

replace order = 15 if measure=="Baroreflex Slope (ms/mmHg)"

* calculate the Meta Analysis with specifies a random-effects model using the DerSimonian and Laird method,

* with the estimate of heterogeneity being taken from the inverse-variance fixed-effect model.

* useb ==1 means that the variable use (version b) must be on (selctetion in ecxeltable wich parameter to be reported

* e.g. if RMSSD and HFpower were available, useb inidcates the selected HRV-parameter

#delimit ;

metan novulatory movulatory sdovulatory nmenses mmenses sdmenses if useb==1, randomi

xlabel( -1 ,0 , 1, 2) force

xtick( -0.5 ,0.5, 1.5, 2.5)

sortby(order condition firstauthor)

astext(80)

textsize(110)

lcols(firstauthor measure position condition)

boxopt(mcolor(gs13))

diamopt(lcolor(navy))

pointopt( msymbol(T) mcolor(navy) msize(tiny))

ciopt( lcolor(gs8) lwidth(medium) )

olineopt(lcolor(navy) lpattern(dash))

favours("Decrease in VA # Increase in VA")

title("Vagal Activity (VA) Change from Menses to Ovulatory Phase", size(small))

graphregion(fcolor(white) lcolor(gs15) ifcolor(white) ilcolor(white)) ;

#delimit cr

graph save "2.2 Menses_ovulatory.gph" , replace

graph export "2.2 Menses_ovulatory.png", replace

# 5. Menstrual vs. Early-to-mid Luteal Phase Comparison

*change the current working directory to the specified drive and directory and import files

cd "[insert your path here]"

import excel " jcm-618911-Supplemental Materials 2.xlsx", sheet("2 Menses vs. Luteal") firstrow clear case(lower)

destring _all , replace

d

***calculate SD from SE

*-*

*Not necessary for 2.2

*set order of HRV parameters in Graph

tab measure,m

gen order =.

replace order = 1 if measure=="RMSSD"

replace order = 2 if measure=="HF"

replace order = 3 if measure=="lnHF"

replace order = 4 if measure=="HFnu"

replace order = 5 if measure=="HF in sec²"

replace order = 6 if measure=="HF m/sn²"

replace order = 7 if measure=="HF power (%)"

replace order = 8 if measure=="HF power in arbitrary units"

replace order = 8 if measure=="HRR"

replace order = 10 if measure=="HRR%"

replace order = 11 if measure=="CVI"

replace order = 12 if measure=="DBT"

replace order = 13 if measure=="Lying-Standing Ratio"

replace order = 14 if measure=="Operational Point (%)"

replace order = 15 if measure=="Valsalva"

replace order = 15 if measure=="Baroreflex Slope (ms/mmHg)"

* calculate the Meta Analysis with specifies a random-effects model using the DerSimonian and Laird method,

* with the estimate of heterogeneity being taken from the inverse-variance fixed-effect model.

* useb ==1 means that the variable use (version b) must be on (selctetion in ecxeltable wich parameter to be reported

* e.g. if RMSSD and HFpower were available, useb inidcates the selected HRV-parameter

#delimit ;

metan nluteal mluteal sdluteal nmenses mmenses sdmenses if useb==1, randomi

xlabel( -1 ,0 , 1, 2) force

xtick( -0.5 ,0.5, 1.5, 2.5)

sortby(order condition firstauthor)

astext(80)

textsize(160)

lcols(firstauthor measure position condition)

boxopt(mcolor(gs13))

diamopt(lcolor(navy))

pointopt( msymbol(T) mcolor(navy) )

ciopt( lcolor(gs8) lwidth(medium) )

olineopt(lcolor(navy) lpattern(dash))

favours("Decrease in VA # Increase in VA")

title("Vagal Activity (VA) Change from Menses to Luteal Phase", size(small))

graphregion(fcolor(white) lcolor(gs15) ifcolor(white) ilcolor(white)) ;

#delimit cr

graph save "2.3 Menses_Luteal.gph" , replace

graph export "2.3 Menses_Luteal.png", replace

# 6. Menstrual vs. Premenstrual Phase Comparison

*change the current working directory to the specified drive and directory and import files

cd "[insert your path here]"

import excel " jcm-618911-Supplemental Materials 2.xlsx", sheet("2 Menses vs. Premenstrual") firstrow clear case(lower)

destring _all , replace

d

***calculate SD from SE

replace sdmenses = sqrt(nmenses)*sdmenses if cavesevssdmeanvsmedian=="SE instead of SD"

replace sdpremenstrual = sqrt(npremenstrual)*sdpremenstrual if cavesevssdmeanvsmedian=="SE instead of SD"

*set order of HRV parameters in Graph

tab measure,m

gen order =.

replace order = 1 if measure=="RMSSD"

replace order = 2 if measure=="logRMSSD"

replace order = 3 if measure=="HF in msec²"

replace order = 4 if measure=="HF in sec²"

replace order = 5 if measure=="HF in m/sn²"

replace order = 6 if measure=="lnHF"

replace order = 7 if measure=="HFnu"

replace order = 8 if measure=="HF power (%)"

replace order = 9 if measure=="HF power in arbitrary units"

replace order = 10 if measure=="HRR"

replace order = 11 if measure=="HRR%"

replace order = 12 if measure=="CVI"

replace order = 13 if measure=="DBT"

replace order = 14 if measure=="Lying-Standing Ratio"

replace order = 15 if measure=="Operational Point (%)"

replace order = 16 if measure=="Valsalva"

replace order = 17 if measure=="BS (ms/mmHg)"

lab var firstauthor "Author(Year)"

lab var withinperson "Within Person"

lab var measure "Measure"

* calculate the Meta Analysis with specifies a random-effects model using the DerSimonian and Laird method,

* with the estimate of heterogeneity being taken from the inverse-variance fixed-effect model.

* useb ==1 means that the variable use (version b) must be on (selctetion in ecxeltable wich parameter to be reported

* e.g. if RMSSD and HFpower were available, useb inidcates the selected HRV-parameter

#delimit ;

metan npremenstrual mpremenstrual sdpremenstrual nmenses mmenses sdmenses if useb==1, randomi

xlabel(-5, -4, -3, -2, -1 ,0 , 1) force

xtick( -5.5, -4.5, -3.5, -2.5, -1.5, -0.5 ,0.5)

sortby(order firstauthor condition)

astext(80)

textsize(110)

lcols(firstauthor measure position condition)

boxopt(mcolor(gs13))

diamopt(lcolor(navy))

pointopt( msymbol(T) mcolor(navy) msize(tiny))

ciopt( lcolor(gs8) lwidth(medium) )

olineopt(lcolor(navy) lpattern(dash))

favours("Decrease in CVA # Increase in CVA")

title("Cardiac vagal activity (CVA) change from menses to premenstrual phase", size(small))

graphregion(fcolor(white) lcolor(gs15) ifcolor(white) ilcolor(white)) ;

#delimit cr

graph save "2.4 Menses_premenstrual.gph" , replace

graph export "2.4 Menses_premenstrual.png", replace

ex

#delimit ;

metan npremenstrual mpremenstrual sdpremenstrual nmenses mmenses sdmenses if usec==1, randomi

xlabel(-5, -4, -3, -2, -1 ,0 , 1) force

xtick( -5.5, -4.5, -3.5, -2.5, -1.5, -0.5 ,0.5)

sortby(order condition firstauthor)

astext(80)

textsize(110)

lcols(firstauthor measure position condition)

boxopt(mcolor(gs13))

diamopt(lcolor(navy))

pointopt( msymbol(T) mcolor(navy) msize(tiny))

ciopt( lcolor(gs8) lwidth(medium) )

olineopt(lcolor(navy) lpattern(dash))

favours("Decrease in VA # Increase in VA")

title("Vagal Activity (VA) Change from Menses to Premenstrual Phase", size(small))

graphregion(fcolor(white) lcolor(gs15) ifcolor(white) ilcolor(white)) ;

#delimit cr

# 6.1 Menstrual vs. Premenstrual Phase Comparison – PMS/PMDD Subgroups Excluded

*change the current working directory to the specified drive and directory and import files

cd "[insert your path here]"

import excel " jcm-618911-Supplemental Materials 2.xlsx", sheet("2 Men vs. Premen OHNE PMS PMDD") firstrow clear case(lower)

destring _all , replace

d

*calculate SD from SE

replace sdfollicular = sqrt(nfollicular)*sdfollicular if cavesevssdmeanvsmedian=="SE instead of SD"

replace sdpremenstrual = sqrt(npremenstrual)*sdpremenstrual if cavesevssdmeanvsmedian=="SE instead of SD"

*set order of HRV parameters in Graph

tab measure,m

gen order =.

replace order = 1 if measure=="RMSSD"

replace order = 2 if measure=="HF"

replace order = 3 if measure=="lnHF"

replace order = 4 if measure=="HFnu"

replace order = 5 if measure=="HF in sec²"

replace order = 6 if measure=="HF m/sn²"

replace order = 7 if measure=="HF power (%)"

replace order = 8 if measure=="HF power in arbitrary units"

replace order = 8 if measure=="HRR"

replace order = 10 if measure=="HRR%"

replace order = 11 if measure=="CVI"

replace order = 12 if measure=="DBT"

replace order = 13 if measure=="Lying-Standing Ratio"

replace order = 14 if measure=="Operational Point (%)"

replace order = 15 if measure=="Valsalva"

replace order = 15 if measure=="Baroreflex Slope (ms/mmHg)"

* calculate the Meta Analysis with specifies a random-effects model using the DerSimonian and Laird method,

* with the estimate of heterogeneity being taken from the inverse-variance fixed-effect model.

* useb ==1 means that the variable use (version b) must be on (selctetion in ecxeltable wich parameter to be reported

* e.g. if RMSSD and HFpower were available, useb inidcates the selected HRV-parameter

#delimit ;

metan npremenstrual mpremenstrual sdpremenstrual nfollicular mfollicular sdfollicular if useb==1, randomi

xlabel(-5, -4, -3, -2, -1 ,0 , 1, 2) force

xtick( -4.5, -3.5, -2.5, -1.5, -0.5 ,0.5, 1.5)

sortby(order condition firstauthor)

astext(75)

textsize(110)

lcols(firstauthor measure position condition)

boxopt(mcolor(gs13))

diamopt(lcolor(navy))

pointopt( msymbol(T) mcolor(navy) msize(tiny))

ciopt( lcolor(gs8) lwidth(medium) )

olineopt(lcolor(navy) lpattern(dash))

favours("Decrease in VA # Increase in VA")

title("Vagal Activity (VA) Change from Follicular to Premenstrual Phase", size(small))

graphregion(fcolor(white) lcolor(gs15) ifcolor(white) ilcolor(white)) ;

#delimit cr

graph save "2.9 Menstrual_premenstrual_ohnePMDD.gph" , replace

graph export "2.9 Menstrual_premenstrual_ohnePMDD.png", replace

# 7. Mid-to-late Follicular vs. Ovulatory Phase Comparison

The mid-to-late follicular and ovulatory phase could not be meta-analytically compared since this comparisons was only reported by one study (Tenan et al., 2014). Therefore, no syntax is available.

# 8. Mid-to-late Follicular vs. Early-to-mid Luteal Phase Comparison

*change the current working directory to the specified drive and directory and import files

cd "[insert your path here]"

import excel " jcm-618911-Supplemental Materials 2.xlsx", sheet("2 Follicular vs. Luteal") firstrow clear case(lower)

destring _all , replace

tostring cavesevssdmeanvsmedian , replace

d

*calculate SD from SE

replace sdfollicular = sqrt(nfollicular)*sdfollicular if cavesevssdmeanvsmedian=="SE instead of SD"

replace sdluteal = sqrt(nluteal)*sdluteal if cavesevssdmeanvsmedian=="SE instead of SD"

*set order of HRV parameters in Graph

tab measure,m

gen order =.

replace order = 1 if measure=="RMSSD"

replace order = 2 if measure=="HF"

replace order = 3 if measure=="lnHF"

replace order = 4 if measure=="HFnu"

replace order = 5 if measure=="HF in sec²"

replace order = 6 if measure=="HF m/sn²"

replace order = 7 if measure=="HF power (%)"

replace order = 8 if measure=="HF power in arbitrary units"

replace order = 8 if measure=="HRR"

replace order = 10 if measure=="HRR%"

replace order = 11 if measure=="CVI"

replace order = 12 if measure=="DBT"

replace order = 13 if measure=="Lying-Standing Ratio"

replace order = 14 if measure=="Operational Point (%)"

replace order = 15 if measure=="Valsalva"

replace order = 15 if measure=="Baroreflex Slope (ms/mmHg)"

* calculate the Meta Analysis with specifies a random-effects model using the DerSimonian and Laird method,

* with the estimate of heterogeneity being taken from the inverse-variance fixed-effect model.

* useb ==1 means that the variable use (version b) must be on (selctetion in ecxeltable wich parameter to be reported

* e.g. if RMSSD and HFpower were available, useb inidcates the selected HRV-parameter

#delimit ;

metan nluteal mluteal sdluteal nfollicular mfollicular sdfollicular if useb==1, randomi

xlabel( -1 ,0 , 1) force

xtick( -1.5, -0.5 ,0.5)

sortby(order condition firstauthor)

astext(80)

textsize(110)

lcols(firstauthor measure position condition)

boxopt(mcolor(gs13))

diamopt(lcolor(navy))

pointopt( msymbol(T) mcolor(navy) msize(tiny))

ciopt( lcolor(gs8) lwidth(medium) )

olineopt(lcolor(navy) lpattern(dash))

favours("Decrease in VA # Increase in VA")

title("Vagal Activity (VA) Change from Follicular to Luteal Phase", size(small))

graphregion(fcolor(white) lcolor(gs15) ifcolor(white) ilcolor(white)) ;

#delimit cr

graph save "2.5 Follicular_luteal.gph" , replace

graph export "2.5 Follicular_luteal.png", replace

# 9. Mid-to-late Follicular vs. Premenstrual Phase Comparison

*change the current working directory to the specified drive and directory and import files

cd "[insert your path here]"

import excel " jcm-618911-Supplemental Materials 2.xlsx", sheet("2 Follicular vs. Premenstrual") firstrow clear case(lower)

destring _all , replace

d

*calculate SD from SE

replace sdfollicular = sqrt(nfollicular)*sdfollicular if cavesevssdmeanvsmedian=="SE instead of SD"

replace sdpremenstrual = sqrt(npremenstrual)*sdpremenstrual if cavesevssdmeanvsmedian=="SE instead of SD"

*set order of HRV parameters in Graph

tab measure,m

gen order =.

replace order = 1 if measure=="RMSSD"

replace order = 2 if measure=="log RMSSD"

replace order = 3 if measure=="HF in msec²"

replace order = 4 if measure=="HF in sec²"

replace order = 5 if measure=="HF in m/sn²"

replace order = 6 if measure=="lnHF"

replace order = 7 if measure=="HFnu"

replace order = 8 if measure=="HF power (%)"

replace order = 9 if measure=="HF power in arbitrary units"

replace order = 10 if measure=="HRR"

replace order = 11 if measure=="HRR%"

replace order = 12 if measure=="CVI"

replace order = 13 if measure=="DBT"

replace order = 14 if measure=="Lying-Standing Ratio"

replace order = 15 if measure=="Operational Point (%)"

replace order = 16 if measure=="Valsalva"

replace order = 17 if measure=="BS (ms/mmHg)"

lab var firstauthor "Author(Year)"

lab var withinperson "Within Person"

lab var measure "Measure"

* calculate the Meta Analysis with specifies a random-effects model using the DerSimonian and Laird method,

* with the estimate of heterogeneity being taken from the inverse-variance fixed-effect model.

* useb ==1 means that the variable use (version b) must be on (selctetion in ecxeltable wich parameter to be reported

* e.g. if RMSSD and HFpower were available, useb inidcates the selected HRV-parameter

#delimit ;

metan npremenstrual mpremenstrual sdpremenstrual nfollicular mfollicular sdfollicular if useb==1, randomi

xlabel(-5, -4, -3, -2, -1 ,0 , 1, 2) force

xtick( -4.5, -3.5, -2.5, -1.5, -0.5 ,0.5, 1.5)

sortby(order condition firstauthor)

astext(75)

textsize(110)

lcols(firstauthor measure position condition)

boxopt(mcolor(gs13))

diamopt(lcolor(navy))

pointopt( msymbol(T) mcolor(navy) msize(tiny))

ciopt( lcolor(gs8) lwidth(medium) )

olineopt(lcolor(navy) lpattern(dash))

favours("Decrease in CVA # Increase in CVA")

title("Cardiac vagal activity (CVA) change from follicular to premenstrual phase", size(small))

graphregion(fcolor(white) lcolor(gs15) ifcolor(white) ilcolor(white)) ;

#delimit cr

graph save "2.6 Follicular_premenstrual.gph" , replace

graph export "2.6 Follicular_premenstrual.png", replace

# 9.1 Mid-to-late Follicular vs. Premenstrual Phase Comparison – PMS/PMDD Subgroups Excluded

*change the current working directory to the specified drive and directory and import files

cd "[insert your path here]"

import excel " jcm-618911-Supplemental Materials 2.xlsx", sheet("2 Foll vs. Premen OHNE PMS PMDD") firstrow clear case(lower)

destring _all , replace

d

*calculate SD from SE

replace sdfollicular = sqrt(nfollicular)*sdfollicular if cavesevssdmeanvsmedian=="SE instead of SD"

replace sdpremenstrual = sqrt(npremenstrual)*sdpremenstrual if cavesevssdmeanvsmedian=="SE instead of SD"

*set order of HRV parameters in Graph

tab measure,m

gen order =.

replace order = 1 if measure=="RMSSD"

replace order = 2 if measure=="HF"

replace order = 3 if measure=="lnHF"

replace order = 4 if measure=="HFnu"

replace order = 5 if measure=="HF in sec²"

replace order = 6 if measure=="HF m/sn²"

replace order = 7 if measure=="HF power (%)"

replace order = 8 if measure=="HF power in arbitrary units"

replace order = 8 if measure=="HRR"

replace order = 10 if measure=="HRR%"

replace order = 11 if measure=="CVI"

replace order = 12 if measure=="DBT"

replace order = 13 if measure=="Lying-Standing Ratio"

replace order = 14 if measure=="Operational Point (%)"

replace order = 15 if measure=="Valsalva"

replace order = 15 if measure=="Baroreflex Slope (ms/mmHg)"

* calculate the Meta Analysis with specifies a random-effects model using the DerSimonian and Laird method,

* with the estimate of heterogeneity being taken from the inverse-variance fixed-effect model.

* useb ==1 means that the variable use (version b) must be on (selctetion in ecxeltable wich parameter to be reported

* e.g. if RMSSD and HFpower were available, useb inidcates the selected HRV-parameter

#delimit ;

metan npremenstrual mpremenstrual sdpremenstrual nfollicular mfollicular sdfollicular if useb==1, randomi

xlabel(-5, -4, -3, -2, -1 ,0 , 1, 2) force

xtick( -4.5, -3.5, -2.5, -1.5, -0.5 ,0.5, 1.5)

sortby(order condition firstauthor)

astext(75)

textsize(110)

lcols(firstauthor measure position condition)

boxopt(mcolor(gs13))

diamopt(lcolor(navy))

pointopt( msymbol(T) mcolor(navy) msize(tiny))

ciopt( lcolor(gs8) lwidth(medium) )

olineopt(lcolor(navy) lpattern(dash))

favours("Decrease in VA # Increase in VA")

title("Vagal Activity (VA) Change from Follicular to Premenstrual Phase", size(small))

graphregion(fcolor(white) lcolor(gs15) ifcolor(white) ilcolor(white)) ;

#delimit cr

graph save "2.6 Follicular_premenstrual_ohnePMDD.gph" , replace

graph export "2.6 Follicular_premenstrual_ohnePMDD.png", replace

# 10. Ovulatory vs. Early-to-mid Luteal Phase Comparison

*change the current working directory to the specified drive and directory and import files

cd "[insert your path here]"

import excel " jcm-618911-Supplemental Materials 2.xlsx", sheet("2 Ovulatory vs. Luteal") firstrow clear case(lower)

destring _all , replace

d

*calculate SD from SE

* not needed

*set order of HRV parameters in Graph

tab measure,m

gen order =.

replace order = 1 if measure=="RMSSD"

replace order = 2 if measure=="HF"

replace order = 3 if measure=="lnHF"

replace order = 4 if measure=="HFnu"

replace order = 5 if measure=="HF in sec²"

replace order = 6 if measure=="HF m/sn²"

replace order = 7 if measure=="HF power (%)"

replace order = 8 if measure=="HF power in arbitrary units"

replace order = 8 if measure=="HRR"

replace order = 10 if measure=="HRR%"

replace order = 11 if measure=="CVI"

replace order = 12 if measure=="DBT"

replace order = 13 if measure=="Lying-Standing Ratio"

replace order = 14 if measure=="Operational Point (%)"

replace order = 15 if measure=="Valsalva"

replace order = 15 if measure=="Baroreflex Slope (ms/mmHg)"

* calculate the Meta Analysis with specifies a random-effects model using the DerSimonian and Laird method,

* with the estimate of heterogeneity being taken from the inverse-variance fixed-effect model.

* useb ==1 means that the variable use (version b) must be on (selctetion in ecxeltable wich parameter to be reported

* e.g. if RMSSD and HFpower were available, useb inidcates the selected HRV-parameter

#delimit ;

metan nluteal mluteal sdluteal novulatory movulatory sdovulatory if useb==1, randomi

xlabel( -1 ,0 , 1) force

xtick( -0.5 ,0.5)

sortby(order condition firstauthor)

astext(80)

textsize(140)

lcols(firstauthor measure position condition)

boxopt(mcolor(gs13))

diamopt(lcolor(navy))

pointopt( msymbol(T) mcolor(navy) msize(tiny))

ciopt( lcolor(gs8) lwidth(medium) )

olineopt(lcolor(navy) lpattern(dash))

favours("Decrease in VA # Increase in VA")

title("Vagal Activity (VA) Change from Ovulatory to Luteal Phase", size(small))

graphregion(fcolor(white) lcolor(gs15) ifcolor(white) ilcolor(white)) ;

#delimit cr

graph save "2.7 ovulatory_luteal.gph" , replace

graph export "2.7 ovulatory_luteal.png", replace

# 11. Ovulatory vs. Premenstrual Phase Comparison

The ovulatory and premenstrual phase could not be meta-analytically compared since this comparison was only reported by one study (Tenan et al., 2014). Therefore, no syntax is available.

# 12. Early-to-mid Luteal vs. Premenstrual Phase Comparison

*change the current working directory to the specified drive and directory and import files

cd "[insert your path here]"

import excel " jcm-618911-Supplemental Materials 2.xlsx", sheet("2 Luteal vs. Premenstrual") firstrow clear case(lower)

destring _all , replace

d

*calculate SD from SE

* not needed

*set order of HRV parameters in Graph

tab measure,m

gen order =.

replace order = 1 if measure=="RMSSD"

replace order = 2 if measure=="HF"

replace order = 3 if measure=="lnHF"

replace order = 4 if measure=="HFnu"

replace order = 5 if measure=="HF in sec²"

replace order = 6 if measure=="HF m/sn²"

replace order = 7 if measure=="HF power (%)"

replace order = 8 if measure=="HF power in arbitrary units"

replace order = 8 if measure=="HRR"

replace order = 10 if measure=="HRR%"

replace order = 11 if measure=="CVI"

replace order = 12 if measure=="DBT"

replace order = 13 if measure=="Lying-Standing Ratio"

replace order = 14 if measure=="Operational Point (%)"

replace order = 15 if measure=="Valsalva"

replace order = 15 if measure=="Baroreflex Slope (ms/mmHg)"

* calculate the Meta Analysis with specifies a random-effects model using the DerSimonian and Laird method,

* with the estimate of heterogeneity being taken from the inverse-variance fixed-effect model.

* useb ==1 means that the variable use (version b) must be on (selctetion in ecxeltable wich parameter to be reported

* e.g. if RMSSD and HFpower were available, useb inidcates the selected HRV-parameter

#delimit ;

metan npremenstrual mpremenstrual sdpremenstrual nluteal mluteal sdluteal if useb==1, randomi

xlabel( -1 ,0 , 1) force

xtick( -0.5 ,0.5, 1.5)

sortby(order condition firstauthor)

astext(75)

textsize(140)

lcols(firstauthor measure position condition)

boxopt(mcolor(gs13))

diamopt(lcolor(navy))

pointopt( msymbol(T) mcolor(navy) msize(tiny))

ciopt( lcolor(gs8) lwidth(medium) )

olineopt(lcolor(navy) lpattern(dash))

favours("Decrease in VA # Increase in VA")

title("Vagal Activity (VA) Change from Luteal to Premenstrual Phase", size(small))

graphregion(fcolor(white) lcolor(gs15) ifcolor(white) ilcolor(white)) ;

#delimit cr

graph save "2.8 luteal_premenstrual.gph" , replace

graph export "2.8 luteal_premenstrual.png", replace

# 13. Ovulatory vs. Premenstrual Phase Comparison

*change the current working directory to the specified drive and directory and import files

cd "[insert your path here]"

import excel " jcm-618911-Supplemental Materials 2.xlsx", sheet("2 Ovulatory vs. Premenstrual") firstrow clear case(lower)

destring _all , replace

d

*calculate SD from SE

* not needed

*set order of HRV parameters in Graph

tab measure,m

gen order =.

replace order = 1 if measure=="RMSSD"

replace order = 2 if measure=="HF"

replace order = 3 if measure=="lnHF"

replace order = 4 if measure=="HFnu"

replace order = 5 if measure=="HF in sec²"

replace order = 6 if measure=="HF m/sn²"

replace order = 7 if measure=="HF power (%)"

replace order = 8 if measure=="HF power in arbitrary units"

replace order = 8 if measure=="HRR"

replace order = 10 if measure=="HRR%"

replace order = 11 if measure=="CVI"

replace order = 12 if measure=="DBT"

replace order = 13 if measure=="Lying-Standing Ratio"

replace order = 14 if measure=="Operational Point (%)"

replace order = 15 if measure=="Valsalva"

replace order = 15 if measure=="Baroreflex Slope (ms/mmHg)"

* calculate the Meta Analysis with specifies a random-effects model using the DerSimonian and Laird method,

* with the estimate of heterogeneity being taken from the inverse-variance fixed-effect model.

* useb ==1 means that the variable use (version b) must be on (selctetion in ecxeltable wich parameter to be reported

* e.g. if RMSSD and HFpower were available, useb inidcates the selected HRV-parameter

#delimit ;

metan npremenstrual mpremenstrual sdpremenstrual novulatory movulatory sdovulatory if useb==1, randomi

xlabel( -1 ,0 , 1) force

xtick( -0.5 ,0.5, 1.5)

sortby(order condition firstauthor)

astext(75)

textsize(140)

lcols(firstauthor measure position condition)

boxopt(mcolor(gs13))

diamopt(lcolor(navy))

pointopt( msymbol(T) mcolor(navy) msize(tiny))

ciopt( lcolor(gs8) lwidth(medium) )

olineopt(lcolor(navy) lpattern(dash))

favours("Decrease in VA # Increase in VA")

title("Vagal Activity (VA) Change from Ovulatory to Premenstrual Phase", size(small))

graphregion(fcolor(white) lcolor(gs15) ifcolor(white) ilcolor(white)) ;

#delimit cr

graph save "2 ovulatory_premenstrual.gph" , replace

graph export "2 ovulatory_premenstrual.png", replace
